# DAAM Is Required for Thin Filament Formation and Sarcomerogenesis during Muscle Development in Drosophila

**DOI:** 10.1371/journal.pgen.1004166

**Published:** 2014-02-27

**Authors:** Imre Molnár, Ede Migh, Szilárd Szikora, Tibor Kalmár, Attila G. Végh, Ferenc Deák, Szilvia Barkó, Beáta Bugyi, Zacharias Orfanos, János Kovács, Gábor Juhász, György Váró, Miklós Nyitrai, John Sparrow, József Mihály

**Affiliations:** 1 Institute of Genetics, Biological Research Centre HAS, Szeged, Hungary; 2 Institute of Biophysics, Biological Research Centre HAS, Szeged, Hungary; 3 Institute of Biochemistry, Biological Research Centre HAS, Szeged, Hungary; 4 University of Pécs, Department of Biophysics, Pécs, Hungary; 5 Department of Biology, University of York, York, United Kingdom; 6 Department of Anatomy, Cell and Developmental Biology, Eötvös Loránd University, Budapest, Hungary; 7 Hungarian Academy of Sciences, Office for Subsidized Research Units, Budapest, Hungary; Harvard Medical School, Howard Hughes Medical Institute, United States of America

## Abstract

During muscle development, myosin and actin containing filaments assemble into the highly organized sarcomeric structure critical for muscle function. Although sarcomerogenesis clearly involves the *de novo* formation of actin filaments, this process remained poorly understood. Here we show that mouse and *Drosophila* members of the DAAM formin family are sarcomere-associated actin assembly factors enriched at the Z-disc and M-band. Analysis of dDAAM mutants revealed a pivotal role in myofibrillogenesis of larval somatic muscles, indirect flight muscles and the heart. We found that loss of dDAAM function results in multiple defects in sarcomere development including thin and thick filament disorganization, Z-disc and M-band formation, and a near complete absence of the myofibrillar lattice. Collectively, our data suggest that dDAAM is required for the initial assembly of thin filaments, and subsequently it promotes filament elongation by assembling short actin polymers that anneal to the pointed end of the growing filaments, and by antagonizing the capping protein Tropomodulin.

## Author Summary

Sarcomeres, the smallest contractile units of muscle, are formed by two major filament systems, the myosin containing thick and the actin containing thin filaments. Although it is well established that sarcomerogenesis involves the formation of novel actin filaments, so far it remained largely unclear how these filaments form. In this study, we show that the *Drosophila* and mouse members of the DAAM formin subfamily are sarcomere associated actin assembly factors. Genetic analysis revealed that dDAAM plays an essential role in thin filament formation and sarcomere organization. In addition, we demonstrate that mDaam1 is an early determinant of myofibrillogenesis. Our data suggest that besides a role at the barbed end of the thin filaments, dDAAM also functions at the pointed end where it antagonizes the capping protein Tropomodulin. Based on these observations, we propose that DAAM family formins are very good candidates for being the long sought-after muscle actin nucleators, that also promote filament elongation by assembling short actin polymers that anneal to the Z-disc anchored growing filament. Given that cardiomyopathies, muscular dystrophies and the cardiovascular disease related heart muscle degenerations belong to the major health problems worldwide, understanding the mechanism of how muscles normally form is of immense biomedical relevance.

## Introduction

Striated muscles contain cylindrical structures, myofibrils, composed of repeating elements called sarcomeres, the basic contractile units of muscle. A sarcomere, defined as the region between neighboring Z-discs, contains two filament systems, the actin-containing thin filaments and the myosin II-containing thick filaments, and their associated proteins. The thin filaments are anchored into the Z-disc where they are cross-linked by dimeric α-actinin and a number of other proteins [1]. These filaments extend in both directions from the Z-disc into neighboring sarcomeres. They consist of a filamentous actin (F-actin) core decorated with the regulatory proteins Tropomyosin (TM) and Troponin. Interdigitated with thin filaments are the bipolar thick filaments, composed largely of myosin molecules, that are at the middle of the sarcomere and crosslinked by the M-band proteins. Whereas the structural properties of these macromolecular complexes have been determined in detail in recent decades, much less is known about the *in vivo* assembly of the filaments and Z-discs to form the very regular sarcomeric structures [2]. In particular, the initial assembly of thin filaments and the regulation of actin dynamics during myofibril formation and maintenance remains poorly understood.

Owing to the regular assembly of actin monomers (G-actin) into F-actin, these filaments display a polarized morphology and dynamics with barbed (+) and pointed (−) ends. *In vivo* filament growth likely occurs only at the barbed end, whereas the pointed end is favored for depolymerization [3]. New actin filament formation critically requires a nucleation step, during which a few actin monomers combine to form a nucleation seed, prior to elongation. As nucleation is not favored kinetically, and spontaneous *in vivo* nucleation would lead to anarchic filament assembly, this step is promoted by nucleation factors. Nucleation factors described so far include the Arp 2/3 complex, formins, Spire, Cordon-bleu and Leimodin (Lmod) [4], [5]. Although actin nucleation factors have been extensively studied in many different model systems, the essential nucleation factors in developing muscles have not been clearly identified. Lmod and the mammalian formin Fhod3 have both been implicated in actin assembly in vertebrate striated muscles [6], [7] but subsequent work concluded that they are unlikely to contribute to actin nucleation during the initial stages of myofibril assembly [8], [9], [10], [11]. In fruit flies, the genome harbors no clear Lmod ortholog, and genetic analysis of the *Drosophila* Fhod ortholog, Fhos, and other members of the formin family, such as Diaphanous, Cappuccino or Form3, revealed no clear role in muscle development [12], [13], [14], [15].

Regulation of thin filament elongation and length, thought to be controlled by elongation factors and capping proteins, are also important aspects of actin dynamics in muscles. Elongation factors, such as Ena/VASP proteins or the barbed end binding formins that also function as nucleation factors, promote filament growth, whereas capping protein binding blocks polymerization. In contrast to non-muscle cells where thin filament growth is restricted to the barbed ends, sarcomeric actin filaments elongate from their pointed ends [16]. In each half sarcomere the thin filaments are aligned with the same polarity and their barbed ends are within the Z-discs, where they are capped by CapZ, whereas their pointed ends are capped by Tropomodulin (Tmod). So far Tmod, TM, Lmod and the Sarcomere Length Short (SALS) proteins have all been implicated in thin filament length regulation. Of these, Tmod binding causes thin filament shortening; conversely, loss of Tmod function causes lengthening of actin filaments [16], [17]. TM enhances Tmod binding affinity, whereas Lmod and SALS seem to antagonize the capping activity of Tmod and promote filament elongation from their pointed ends [9], [18]. Surprisingly, instead of promoting elongation, SALS appears to inhibit filament elongation *in vitro*. These results together with the observation that no protein was yet isolated which would catalyze F-actin assembly at the pointed end, mean that the mechanism which enables muscle thin filaments to elongate from their pointed ends remains mysterious.

Here we show that the *Drosophila* formin DAAM (Dishevelled associated activator of morphogenesis) plays an important role in sarcomerogenesis. The absence of *dDAAM* reduces larval motility, causes a flightless phenotype and complex defects in sarcomere organization. The latter include shorter and thinner sarcomeres with reduced thin filament levels and an absence of both Z-disc and M-band organization. Our protein localization studies revealed that, despite being a barbed end binding protein in non-muscle cells, dDAAM is highly enriched near the thin filament pointed ends both in *Drosophila* and mouse muscle cells. We propose that members of the DAAM family of formins are very good candidates for the long sought-after muscle actin/thin filament nucleators.

## Results

### *dDAAM* mutations affect flight ability and IFM development

**Figure 1:**
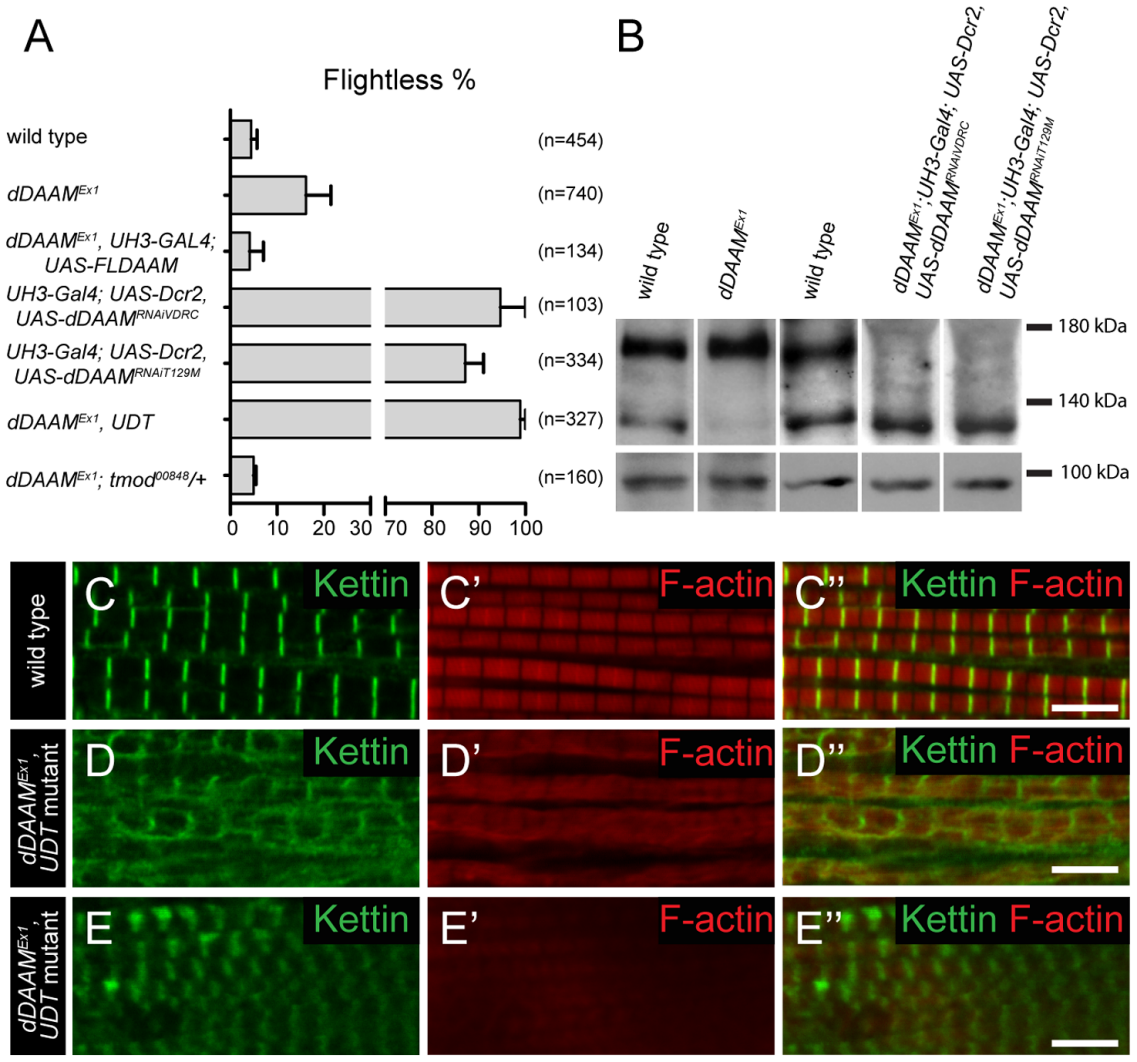
*dDAAM* impairs IFM structure. (A) Quantification of the flight ability of wild type and *dDAAM* mutant flies with the genotypes indicated. Bars display mean±SEM. (B) Western blot shows that wild type IFM expresses two dDAAM protein isoforms, of 130 kD and 163 kD. The larger isoform is more highly expressed of the two. The *dDAAM^Ex1^* allele reduces the level of the 130 kD isoform, whereas RNAi silencing results in a strong reduction of the level of the 163 kD isoform. Lower panel shows the loading control (α-glycogen-phosphorylase). (C–C″) Myofibrils of a wild type IFM display a regular sarcomere organization. (D–E″) Myofibrils from two different IFMs of the *dDAAM^Ex1^*, *UDT* mutant combination. Note the complex sarcomeric defects (D–E″) including the reduced F-actin level (in red, C′–E′), the irregularities in fiber width, the disorganized Z-discs stained with anti-Kettin (in green, C–E″) and the sarcomere length shortening in E–E″. Bars, 5 µm.

In studies of the *Drosophila* formin DAAM, we noticed that ∼16% of adults homozygous for the viable, hypomorphic *dDAAM^Ex1^* allele were flightless (16.2±5.3%, mean±SEM, n = 740, p = 0.02). As *dDAAM* null alleles are homozygous lethal, we used two *dDAAM* specific RNAi lines (*KK102786* from VDRC and *T129M* constructed in our laboratory, targeting two non-overlapping parts of the mRNA) to verify the flight effect. In the presence of *UAS-Dicer2* and an IFM (indirect flight muscle) specific driver (*UH3-Gal4*) [19], both RNAi lines produced strong flightless phenotypes (RNAi^VDRC^: 94.7±5.3%, mean±SEM, n = 103, p<0.001; RNAi^T129M^: 87.1±3.9%, mean±SEM, n = 334, p = 0.002) (Figure 1A). RNAi silencing in a *dDAAM* mutant background (*dDAAM^Ex1^*, *UH3-Gal4*; *UAS-Dicer2*; *UAS-dDAAM^RNAi-T129M^*, subsequently referred to as *dDAAM^Ex1^*, *UDT*) caused nearly all males to be flightless (98.9±1.1%, mean±SEM, n = 327, p<0.001) (Figure 1A). The strength of the flightless phenotypes correlates with the partial reduction of dDAAM protein levels in *dDAAM^Ex1^* IFM and its near absence in IFM from the RNAi genotypes (Figure 1B). The flightless phenotype exhibited by *dDAAM^Ex1^* mutants could be rescued by muscle-specific expression of the dDAAM protein (4.1±2.9%, mean±SEM, n = 134, p = 0.043) (Figure 1A).

In wild type or *UH3-Gal4*; *UAS-Dicer2* flies (used as parental control), the IFM displayed, as visualized by phalloidin (labels F-actin) and anti-Kettin (a Z-disc marker) staining, its typical regular sarcomeric organization (Figure 1C–C″), with the sarcomere length of 3.19±0.04 µm (mean±SD, n = 63) found in young adults. In contrast, the IFM of *dDAAM* mutant flies showed significant structural alterations (Figure 1D–E″). The IFM of flightless *dDAAM^Ex1^* mutants looked largely normal, but about 25% of the myofibrils were thinner (1.42±0.32 µm, mean±SD, n = 50, p<0.001) than wild type (1.72±0.11 µm, mean±SD, n = 150) and part of the sarcomeres exhibited a reduced length (down to 2.59±0.13 µm, mean±SD, n = 73, p<0.001) (Figure S1A). In contrast, IFM from the *dDAAM^Ex1^*, *UDT* mutant combination showed gross alterations in IFM fiber morphology (Figure S3A,B). The myofibrils were thinner than in wild type (1.18±0.3 µm, mean±SD, n = 64, p<0.001) and their organization was irregular (Figure 1D–E″). Mutant IFMs exhibited reduced F-actin staining (Figure 1D–E″) without significant alterations in the amount of G-actin (Figure S1F). Additionally, phalloidin staining suggested that many of the thin filaments were of unequal length, and similar to *dDAAM^Ex1^* mutants, shorter sarcomeres (1.97±0.28 µm, mean±SD, n = 62, p<0.001) could often be detected. M-lines could hardly be identified by Myosin immunostaining (Figure S1B–C″), while the Z-discs displayed a highly irregular and delocalized pattern compared to wild type (Figure 1D–E″). Thus, loss of *dDAAM* function impairs IFM structure from overall muscle shape to myofibrillar and sarcomeric organization.

**Figure 2:**
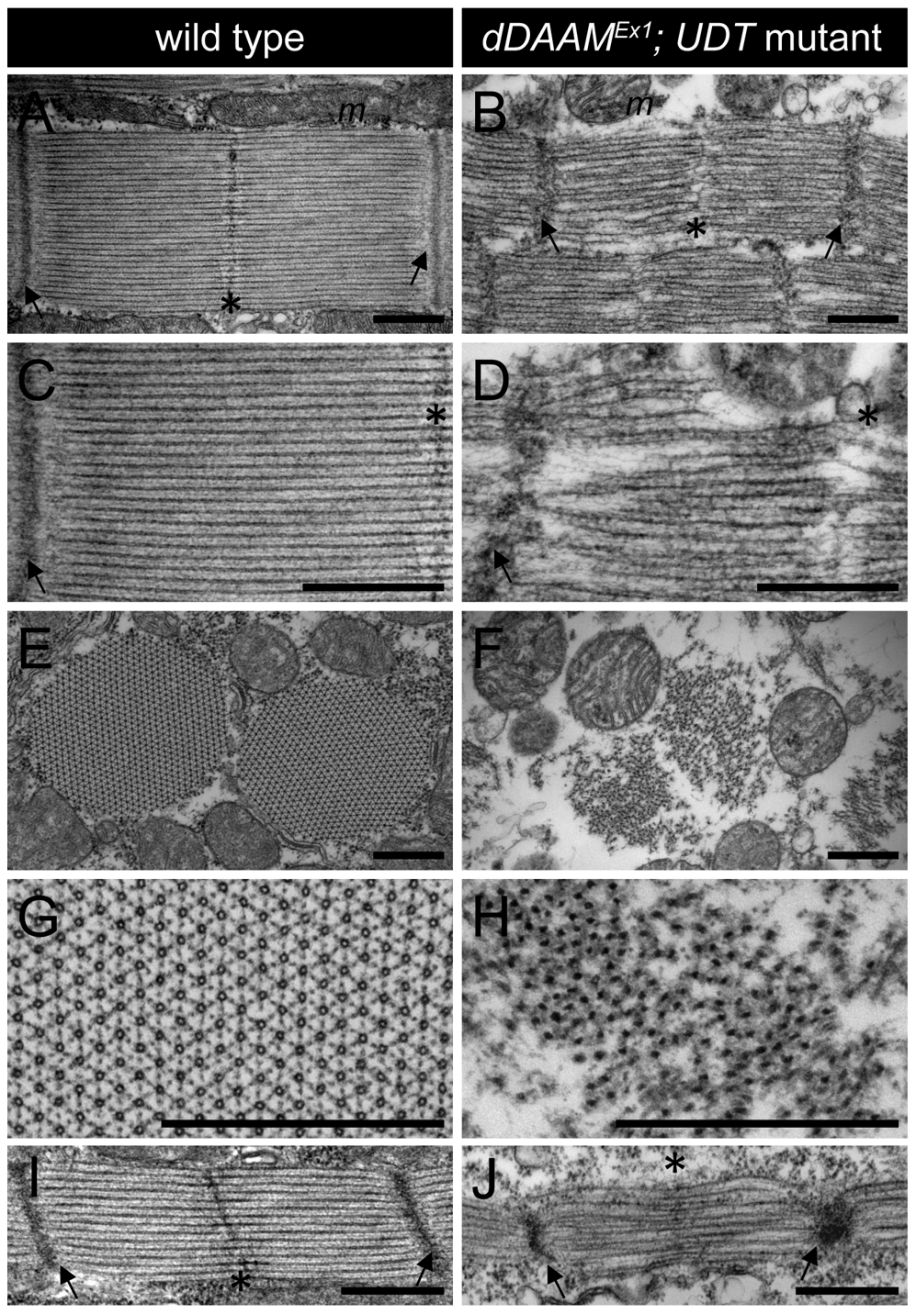
EM analysis of IFM morphology in *dDAAM* mutants. Electronmicrographs of IFM from wild type (A, C, E, G, I) and *dDAAM^Ex1^*; *UDT* mutants (B, D, F, H, J). Longitudinal sections of adult IFM (A–D) show that, as compared to the wild type, highly ordered and tightly packed sarcomeres (A, C), the *dDAAM* mutant myofibrils (B, D) display Z-disc and M-band defects, and shortened sarcomeres with loosely organized thin and thick filaments. Transverse sections of wild type (E, G) muscles reveal the hexagonal lattice organization of thin and thick filaments, which is almost entirely lost in *dDAAM* mutant myofibrils (F, H). Instead, the mutant fibrils are irregularly shaped, consisting of clusters of thick filaments, and individual thin filaments are hardly detectable. Note: wild type thick filaments are hollow (G), while those of the *dDAAM* mutant are very dark, irregularly shaped and almost never hollow (H). Longitudinal sections of pupal IFM (48 hours APF) (I, J) show that, as compared to wild type (I), mutants (J) have strong Z-disc and M-line defects, shorter sarcomeres and irregular filament organisation. Arrows mark the Z-discs, asterisks mark the M-bands, *m* labels the mitochondria. Bars, 500 nm.

Electron microscopy (EM) of the IFM of *dDAAM^Ex1^*, *UDT* mutants (Figure 2) confirmed and extended all the major myofibrillar defects seen in the confocal images. Notably, in longitudinal sections (Figure 2A–D) we revealed irregularly shaped, thin myofibrils with frayed edges, strong Z-disc defects, absence of M-lines and shorter sarcomeres. The thick and thin filament organization was also severely altered. Thick filaments rarely ran parallel to each other, the average thick filament number per sarcomere was strongly reduced compared to controls, and filament packing was much looser than wild type (Figure 2A–D). Most strikingly, instead of thin filaments running in parallel between the myosin filaments (Figure 2C), this space was filled with many thin filaments that formed a meshwork in some places (Figure 2D). Although the organization of these filaments was very different from wild type, their dimensions argue that they are disordered thin filaments. Possibly, but we consider it unlikely, they are connecting filaments. Such filaments containing the Sallimus/Kettin proteins link the Z-disc to the thick filaments in insect flight muscle. The transverse sections of *dDAAM* mutant IFMs confirmed the presence of irregularly shaped myofibrils consisting of thick filament clusters with grayish material in between of them, whereas individual thin filaments could hardly be seen (Figure 2E–H). Clearly the regular myofibrillar lattice was missing. Unlike wild type thick filaments which appear ring-shaped or hollow in transverse sections (except at the level of the M-line) (Figure 2G) [20], *dDAAM* mutant thick filaments were very dark, irregular in shape and almost never hollow (Figure 2H).

At 48 hours after puparium formation (APF) (at 29°C) the pupal IFM of *dDAAM^Ex1^*, *UDT* flies already showed all the muscle phenotypes observed in adults. These include irregular myofibrillar organization, reduced F-actin levels, lack of visible M-lines and disorganized, unequally spaced Z-discs (Figure S2A–B″). Accordingly, EM analysis revealed sarcomere shortening (2.3±0.05 µm, n = 22 in wild type; 1.53±0.08 µm, mean±SD, n = 22 in mutants, p<0.001), absence of M-lines, erratic filament packing and strong Z-disc defects (Figure 2I–J). Together, these data suggest that the IFM phenotypes observed in newly hatched *dDAAM* mutant adults were likely to be a consequence of loss of *dDAAM* function during early muscle development.

To test whether the structural alterations observed in dDAAM mutant myofibrils affect their mechanical properties an Atomic Force Microscope (AFM) was used to measure the transverse elasticity of individual myofibrils in rigor conditions (Figure S2C). The elasticity (Young's modulus) of *dDAAM^Ex1^* and *dDAAM^Ex1^*, *UDT* mutant myofibrils was significantly lower, 6±1.63 kPa (n = 35) and 4±1.24 kPa (n = 15), than that of wild type, 22±4.91 kPa (n = 25).

In summary, the genetic impairment of *dDAAM* function severely affects the structural and mechanical properties of the flight muscles. These results argue that this formin is an important regulator of muscle development affecting multiple aspects of myofibril formation in flies.

### *dDAAM* impairs somatic muscle formation and heart development

**Figure 3:**
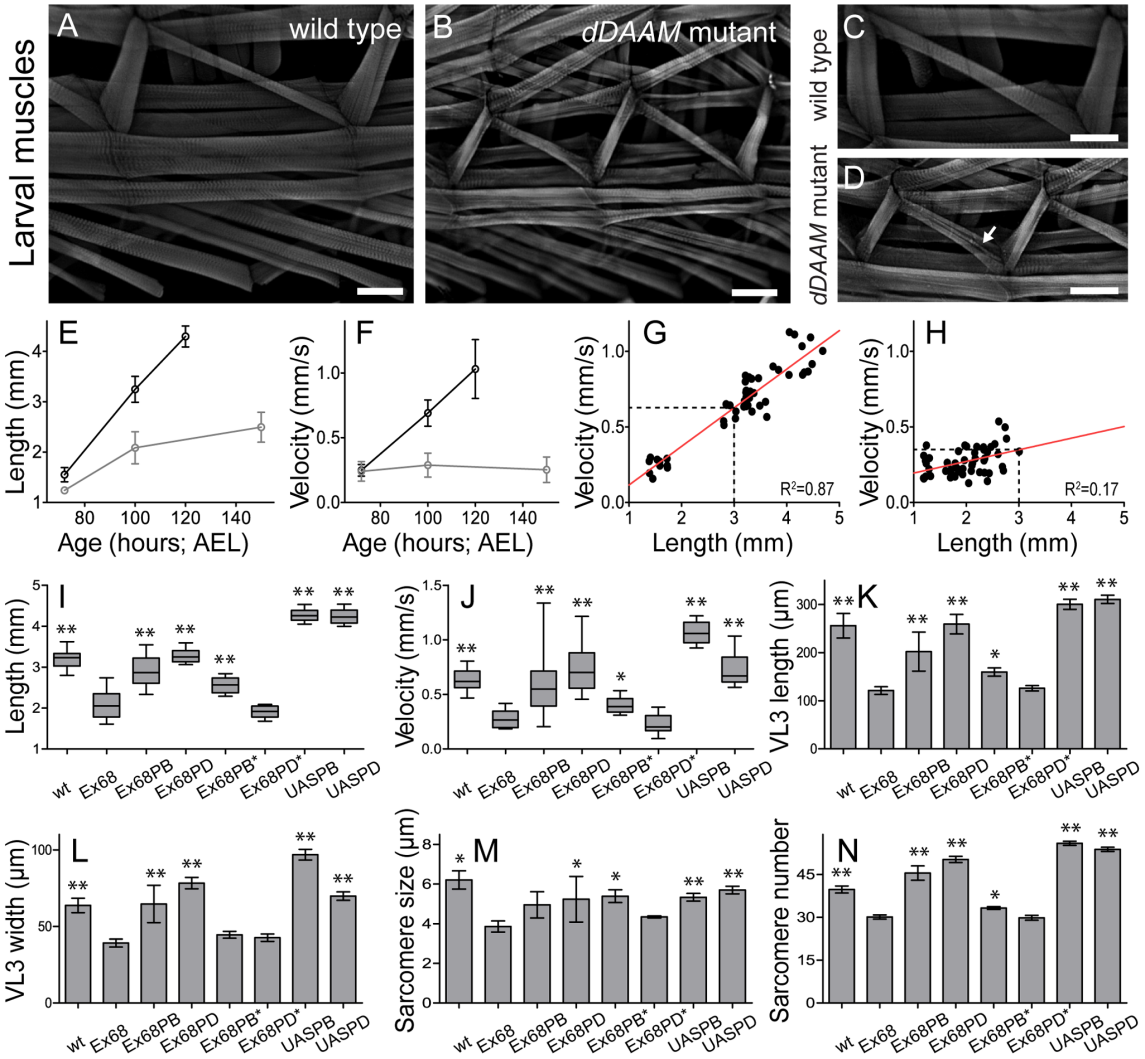
Structural and functional analysis of the larval body wall muscles. Wild type (A, C) and *dDAAM^Ex68^* null mutant (B, D) larval body wall muscles stained with phalloidin. Mutant muscles are smaller, some myofibers are split (arrow on D) and the overall muscle pattern is looser than in wild type. The relationship of larval age and length (E), and of larval age and velocity (F) in *wt* (wild type; black line) and *dDAAM^Ex68^* (grey line) larvae. The relationship of larval length and velocity of *wt* (G) and *dDAAM^Ex68^* mutant (H) larvae. Quantification of larval length (I), crawling velocity (J), VL3 muscle length (K), width (L), mean sarcomere length (M) and serial sarcomere number (N) in larvae 100 hours AEL with the following genotypes: wt (wild type), Ex68 (*dDAAM^Ex68^*), Ex68PB (*dDAAM^Ex68^*; *DMef2-Gal4*; *UAS-dDAAM-PB*), Ex68PD (*dDAAM^Ex68^*; *DMef2-Gal4*; *UAS-dDAAM-PD*), Ex68PB* (*dDAAM^Ex68^*; *DMef2-Gal4*; *UAS-dDAAM-PB^I732A^*), Ex68PD* (*dDAAM^Ex68^*; *DMef2-Gal4*; *UAS-dDAAM-PD^I732A^*), UASPB (*DMef2-Gal4*; *UAS-dDAAM-PB*) and UASPD (*DMef2-Gal4*; *UAS-dDAAM-PD*). Bars represent mean values with respective SDs in I–N. Statistical significance: * 0.05>p<0.001; ** p≤0.001. Wild type and rescue data were compared to *dDAAM^Ex68^* data, unless otherwise indicated in the text. Bars, 100 µm (A–D).

To ask whether dDAAM plays a role generally in muscle development, larval body wall muscles and the heart tube were examined. The body size and somatic musculature of *dDAAM^Ex68^* null mutant early third instar (L3) appeared normal, but late in L3, 100 hours after eggs laying (AEL), the larvae were shorter (2.08±0.31 mm; n = 30) than wild type (3.24±0.25 mm; n = 30; p<0.001; Figure 3E,I). Although gross alterations were not evident in the overall structure of the musculature, mutant muscles were also smaller, some myofibers were split and their general organization was looser than in wild type (Figure 3A–D). Measurements of the ventral longitudinal 3 (VL3) muscle showed a 53% length reduction and 38% reduction in width (Figure 3K,L) compared to wild type. Shortening of VL3 in *dDAAM* mutants arises both by sarcomere shortening and a reduction in sarcomere numbers (Figure 3M,N). The mean sarcomere length of wild type VL3 muscles at 100 hours AEL was 6.2±1.6 µm (n = 477 sarcomeres; 12 muscles), but was decreased in *dDAAM* mutants to 3.8±0.7 µm (n = 241 sarcomeres; 8 muscles; p<0.001). The serial sarcomere number of VL3 was also decreased in *dDAAM* mutants (30.1±2.1; n = 8) compared to wild type (39.7±4.3; n = 12; p<0.001).

To investigate the physiological relevance of the muscular defects observed, we examined the larval motility of *dDAAM* mutant larvae. Until the early L3 stages there were no differences between the wild type and the *dDAAM* mutant larvae, possibly due to maternally derived dDAAM (in ∼10% of *dDAAM^Ex68^* larvae the dDAAM protein could still be clearly detected at 100 hours AEL, Figure S3E,F). Consistent with the findings of the structural analysis, kinematic studies of linear larval crawling at 72 hours AEL showed that velocities of wild type and mutant larvae did not significantly differ (Figure 3F). Subsequently at 100 hours AEL their velocity was decreased by ∼60% compared to wild type (Figure 3F,J). Although, we observed a strong correlation between larval body length and crawling velocity (Figure 3G,H), the *dDAAM* mutant larvae are much slower than their reduced size would indicate. Rescue experiments with *DMef2-Gal4* driven expression of *UAS-DAAM* constructs confirmed that the observed phenotypes are specific to loss of *dDAAM* function. Western blot analysis revealed that the IFM expresses two dDAAM protein isoforms, a short (130 kD) minor isoform and a long (163 kD) major isoform (Figure 1B). These correspond respectively to the predicted DAAM-PB and DAAM-PD proteins (Flybase annotation). The rescue experiments (above) were performed with *UAS-DAAM-PB* as well as with *UAS-DAAM-PD*. *UAS-DAAM-PB* expression partly rescued the velocity decrease and almost fully rescued the body and muscle size of *dDAAM^Ex68^* mutant larvae, whereas *UAS-DAAM-PD* expression almost completely rescued all the phenotypic traits (Figure 3I–N). Moreover, muscle-specific expression of these constructs not only rescued the larval muscle defects, but partly rescued the lethality of *dDAAM^Ex68^* to adulthood (3% for PB and 6.1% for PD). Importantly, unlike the wild type constructs, the actin polymerization incompetent mutant forms, *UAS-DAAM-PB^I732A^* and *UAS-DAAM-PD^I1042A^* mimicking the Bni1 I1431A mutation [21], failed to rescue (Figure 3I–N). These data demonstrate that the effect of *dDAAM* on muscle structure and larval motility is muscle autonomous, and that the actin-assembling activity of dDAAM is essential for normal muscle development. Additionally, it appears that the two muscle-specific dDAAM isoforms play largely, but not completely, redundant roles in larval muscle.

Muscle-specific expression of *UAS-DAAM-PB* and *UAS-DAAM-PD*, in a wild type background, produced significantly longer larvae (PB: 4.26±0.15 mm, n = 10, p<0.001; PD: 4.24±0.19 mm, n = 10, p<0.001) than wild type. Their VL3 muscles were longer, although in both cases sarcomere size was slightly shorter than wild type (Figure 3I,K,M). Muscle lengthening occurred by significantly increasing sarcomere number compared to wild type (PB: 56±2.8, n = 14, p<0.001; PD: 54±2.5, n = 12, p<0.001) (Figure 3N). Interestingly, the aforementioned structural aspects were almost identical in larvae overexpressing either isoform. Nevertheless, larvae expressing the PB isoform were much faster (∼55% faster, n = 10) than wild type larvae (Figure 3J), while the velocity of larvae expressing PD (∼5% faster, n = 10) and the controls (Figure 3J) were not significantly different. Lengths of PB and PD overexpressing larvae were indistinguishable but PB larvae had significantly wider VL3 muscles compared to PD larvae. Thus increasing dDAAM isoform levels is sufficient to enhance the number of sarcomeres initiated, but efficient sarcomere elongation may require cooperation of both isoforms and regulation of their ratio.

Larval heart tube size was also reduced in *dDAAM* mutants compared to wild type (∼40% reduction in diameter). In 100 hour old wild type larvae the maximum heart diameter was 100.33±7.39 µm; n = 9 whereas in *dDAAM* mutants 60.44±6.18 µm; n = 9, p<0.001 and they displayed reduced F-actin levels (Figure S3C,D). Many mutant myofibrils appeared thinner than in wild type and often deviated from the normal orientation (Figure S3D). These observations strongly suggest that the formin dDAAM may be a crucial regulator of muscle development in *Drosophila* with an effect in every muscle type and developmental stage examined.

### Sarcomeric localization of dDAAM

**Figure 4:**
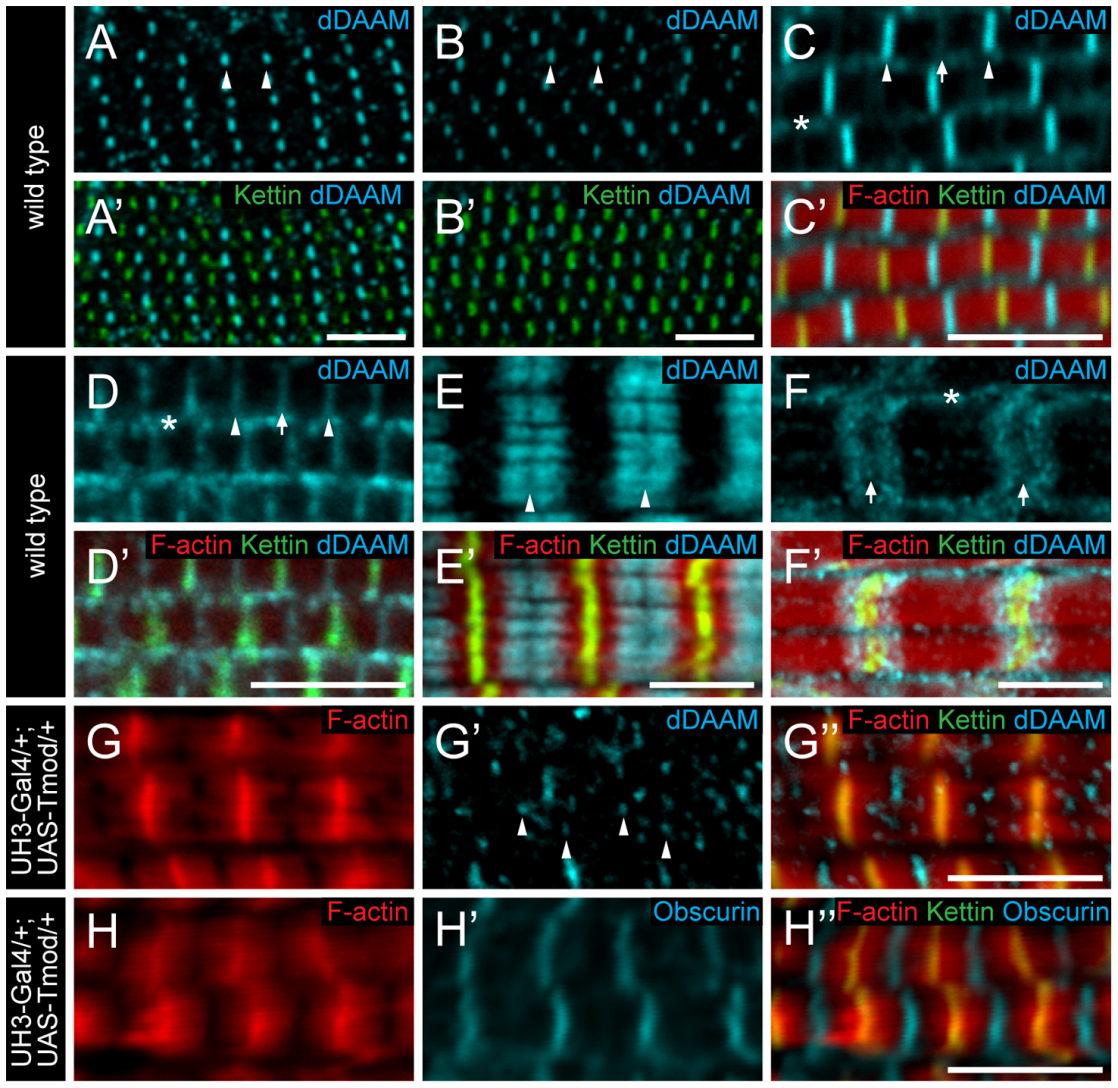
Sarcomeric localization of the dDAAM protein in the IFM and the larval body wall muscles. *dDAAM* staining of the IFM myofibrils of wild type pupae 48 hours (A, A′) and 72 hours APF (B, B′), freshly eclosed adult (C, C′) and 4 day-old adult (D, D′). dDAAM accumulates at the M-line (arrowheads), at the Z-disc (arrow) and in the sarcoplasm (asterisk). Note: accumulation at Z-disc is weak in pupae and young adults (A–C), but in 4 day-old adults staining is equally strong at the M-line and the Z-disc (D). In developing larval body wall muscles (72 hours AEL) dDAAM staining resolves into two bands along the M-line (E, E′). In fully matured larval body wall muscles dDAAM staining relocates to a region flanking the Z-disc (F, F′). Arrowheads mark the M-line in E; arrows mark the Z-disc, asterisk marks the sarcoplasm in F. (G–H″) Excess Tmod in *UH3-Gal4/+*; *UAS-Tmod/+* flies leads to shorter thin filaments that are not in perfect register and vary in length as judged by F-actin staining (G, H). In these IFMs dDAAM protein displays a punctate distribution (arrowheads in G′) most of which colocalizes with the pointed end region of the thin filaments (G″). The M-line in these mutant muscles remains nearly intact as judged by Obscurin staining (H′). Phalloidin staining is in red (C′–H″), Kettin (C′–H″) and sls-GFP (A′, B′) as Z-disc markers are in green, anti-dDAAM (A–F′, G′, G″) and anti-Obscurin (H, H′) are in cyan. Bars, 5 µm.

To further characterize the role of dDAAM in myofibril formation, we examined its localization pattern in the IFM. In newly eclosed adults the anti-dDAAM serum [22] produced a strong staining in the middle of IFM sarcomeres in the M-line region and a weaker staining was evident at the Z-disc and within the sarcoplasm (Figure 4C). This pattern persisted from 48 hours APF (the earliest analyzable pupal developmental timepoint) (Figure 4A–B′) until young adulthood. However, in slightly older adults the signal gradually decreased at the M-line and by 4 days after hatching, equally strong signals were detected at the M-line and Z-disc (Figure 4D). In a *dDAAM^Ex1^*, *UDT* mutant, which is nearly protein null for dDAAM (Figure 1B), only background staining was detectable demonstrating the specificity of the antibody (Figure S4B). To complement the immunostaining we created a C-terminally GFP tagged *dDAAM* knock-in allele (*dDAAM^EGFP^*). The *dDAAM^EGFP^* allele is viable and fertile in either homo- or hemizygous states, and expression of this protein is entirely under the control of endogenous regulatory sequences. The dDAAM::EGFP fusion protein displayed a roughly equally strong enrichment at the M-line and Z-disc in young and 4 day-old IFMs (Figure S1D–E′). Thus, although the anti-dDAAM serum detects a partial difference between the early and late dDAAM pattern, which is not seen with *dDAAM^EGFP^* (presumably due to a difference in the accessibility of the native and the EGFP tagged C-termini), both tools confirm that sarcomeric dDAAM protein is present at both the Z-disc and the M-line.

As thin and thick filaments overlap almost entirely in *Drosophila* IFM, it was not possible to determine unambiguously whether dDAAM enrichment in the middle of wild type sarcomeres reflects binding to the M-line or to the thin filament ends that extend close to the M-line. In *UH3-Gal4/+*; *UAS-Tmod/+* mutant flies excess Tmod resulted in shorter thin filaments that were not in perfect register and varied in length (Figure 4G) while M-line organization remained largely normal (Figure 4H), as judged by F-actin and Obscurin staining, respectively. In such IFMs the dDAAM protein no longer formed a distinct band at the M-line. Instead a punctate intra-sarcomeric staining occurred that mostly co-localized with the pointed end region of the actin filaments (Figure 4G–G″). This suggests that the mid-sarcomeric dDAAM enrichment, seen in wild type, is likely to be thin filament binding and not an M-line association. Consistent with this conclusion, in developing larval body wall muscles (72 hours AEL) the dDAAM staining clearly resolves into two bands along the M-line (Figure 4E). Interestingly, in full-grown larval myofibrils the dDAAM staining relocated to a region flanking the Z-disc (Figure 4F), which is similar to the pattern observed for SALS and Tmod [18]. All together these localization data indicate that dDAAM is present in the growing sarcomeres at a location consistent with a role in thin filament regulation.

### Evolutionary conservation

**Figure 5:**
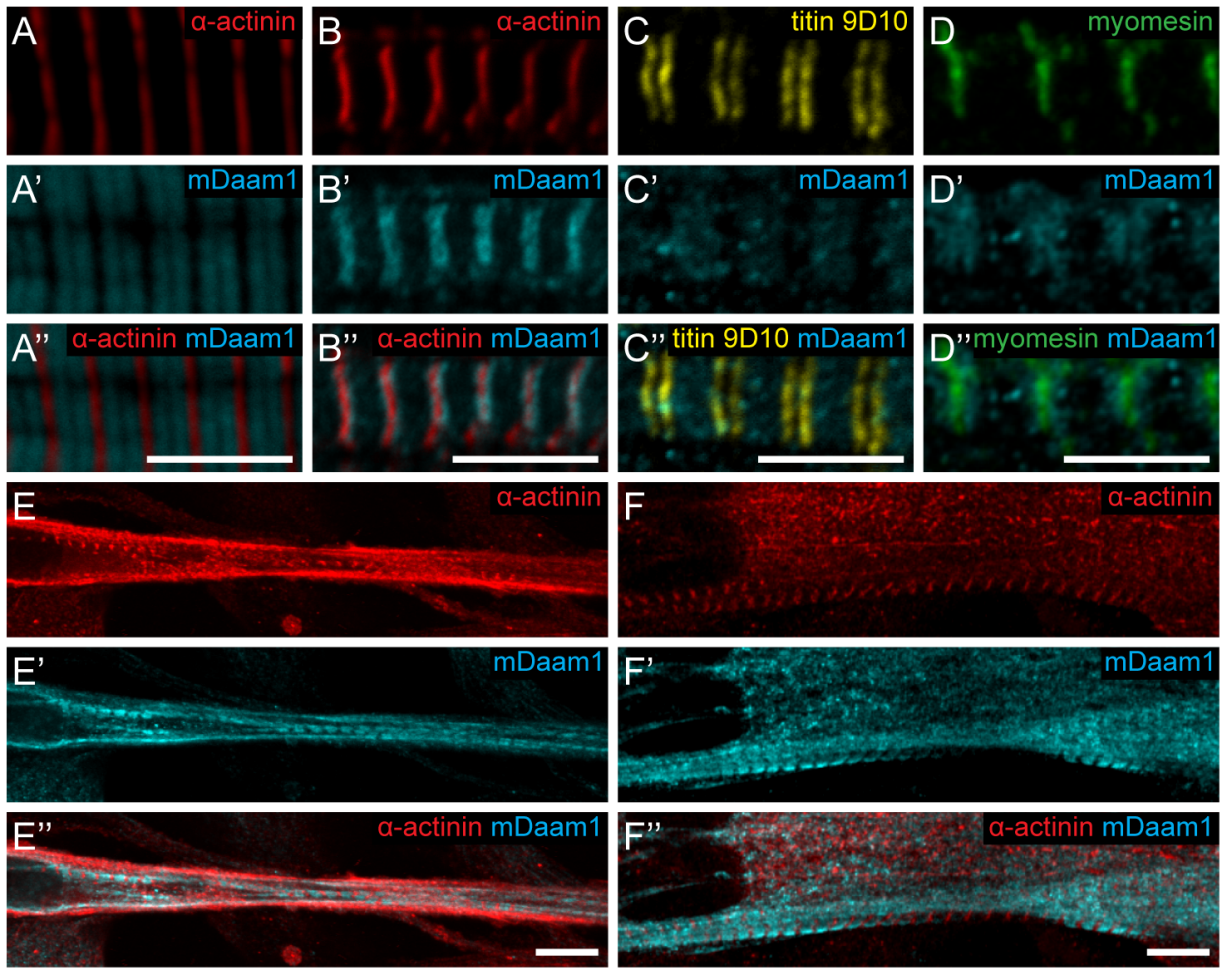
Sarcomeric localization of the mDaam1 protein. (A–B″) mDaam1 staining (in cyan) of mouse muscle sections (the Z-disc marker α-actinin is in red). In *m. tibialis anterior* sarcomeres mDaam1 accumulates in two bands either side of the M-line (A–A″), whereas in *m. vastus lateralis* it is mostly detected along the Z-discs (B–B″). In C_2_C_12_ cells differentiated for 96 hours mDaam1 (cyan) accumulates in two broad bands at the middle of the sarcomere that does not significantly overlap with titin staining (yellow; 9D10 antibody) (C–C″) or myomesin (green), an M-line marker (D–D″). (E–F″) Distribution of mDaam1 (cyan) and α-actinin (red) in C_2_C_12_ cells induced to differentiate for 24 (E–E″) or 96 hours (F–F″). Bars: 5 µm (A–D″); 15 µm (E–F″).

As many muscle proteins are evolutionary highly conserved, and the mouse *Daam1* (*mDaam1*) gene was shown to be involved in heart development [23], we examined the localization of mDaam1 by immunostaining of skeletal muscle sections from 15 day-old animals. Interestingly, in the *m. tibialis anterior* two bands of sarcomeric enrichment occurred at either side of the M-line, whereas in *m. vastus lateralis* most protein was detected along the Z-discs (Figure 5A–B″). To verify this mDaam1 localization pattern further and its development during the early phases of myofibrillogenesis, we used the mouse myogenic cell line C_2_C_12_ [24] and α-actinin, known to be one of the earliest marker of myofibril formation [25]. In C_2_C_12_ cells that were induced to differentiate for 24 hours, mDaam1 was detected in two broad bands in the sarcomeres between the Z-bodies and the M-line (Figure 5E). In C_2_C_12_ cells differentiated for 48 or 96 hours, the same mDaam1 distribution was detected as after 24 hours of differentiation (Figure 5F). To resolve the sarcomeric position of the two bands labeled by anti-mDaam1, double staining was carried out with the anti-titin 9D10 and the anti-myomesin B4 antibodies in C_2_C_12_ cells differentiated for 96 hours. The 9D10 antibody labels the PEVK region of the giant titin protein located in the I-band close to the I-A border [26], [27], whereas B4 labels the M-line [28]. The mDaam1 staining did not significantly overlap with that of either 9D10 or B4 (Figure 5C–D″) confirming that most of the mDaam1 protein is accumulated between the M-line and the I-A border, corresponding to the thin and thick filament overlap region.

The sarcomeric localization pattern of mDaam1 suggests two important conclusions. Firstly, despite some muscle-specific differences, the subsarcomeric localization of mDaam1 appears similar to that of *Drosophila* DAAM with regard to accumulation at the Z-disc and alongside the M-line. Secondly, because mDaam1 is recruited to sarcomeric complexes as early as the actin cross-linker α-actinin protein, this formin is likely to be an early determinant of myofibrillogenesis.

### dDAAM interacts with thin filament mutants

**Figure 6:**
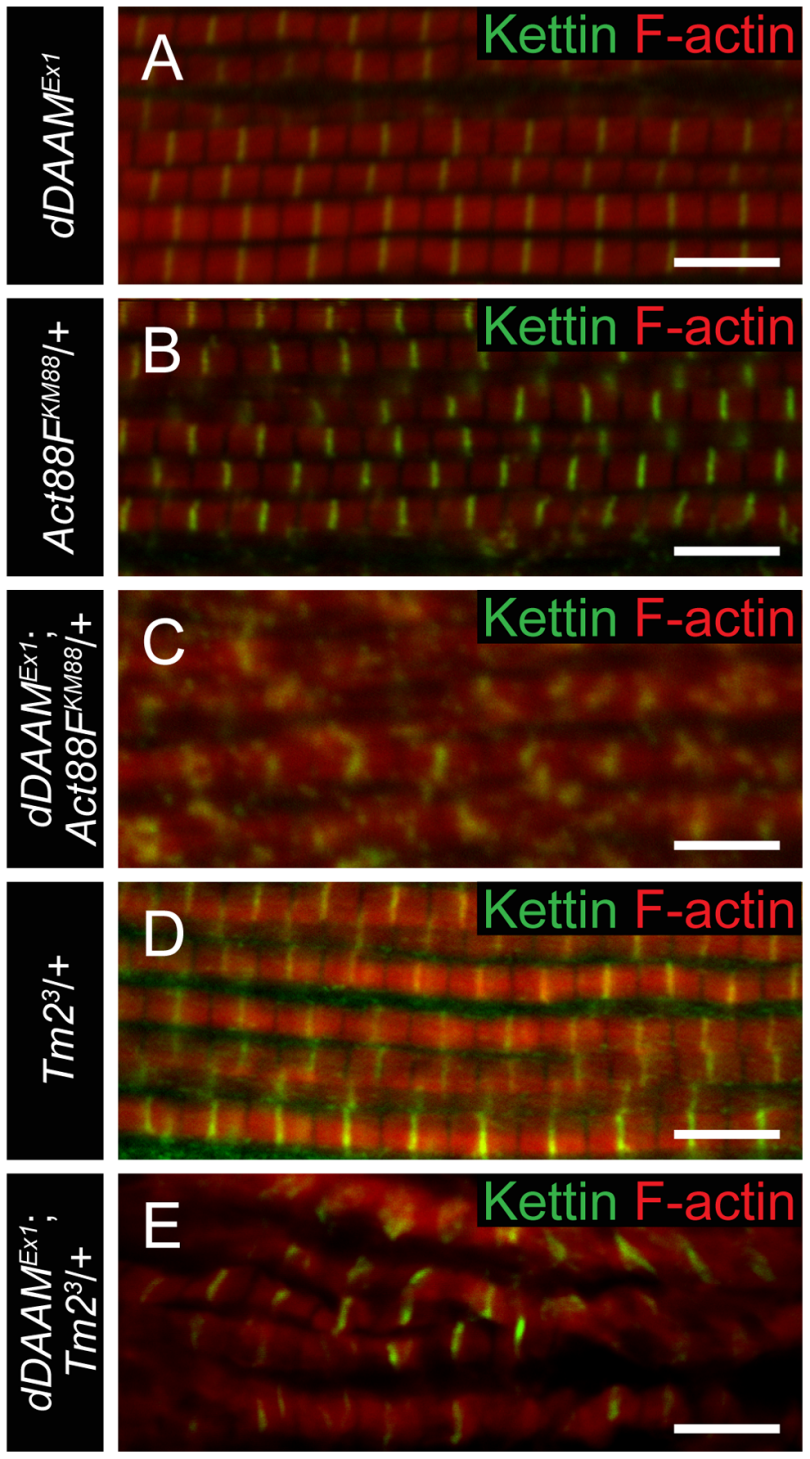
*dDAAM* interacts with thin filament mutants. IFM myofibrils from (A) *dDAAM^Ex1^*, (B) *Act88F^KM88^*/+, (C) *dDAAM^Ex1^*; *Act88F^KM88^*/+, (D) *Tm2^3^*/+ and (E) *dDAAM^Ex1^*; *Tm2^3^*/+ flies (actin in red, Kettin in green in all panels). Note: sarcomere organization in *dDAAM^Ex1^* (A) is nearly wild type; likewise *Act88F* (B) and *Tm2* (D) heterozygotes display a largely regular myofibril and Z-disc organization. Myofibrils of the *dDAAM^Ex1^*; *Act88F^KM88^*/+ (C) and *dDAAM^Ex1^*; *Tm2^3^*/+ (E) genotypes are extremely disorganized compared to the controls. Bars, 5 µm.

To collect further evidence for our proposal that *dDAAM* has an important role in thin filament formation and regulation, genetic interactions with the IFM-specific *Act88F^KM88^* and *Tm2^3^* mutations [29], [30] were tested. IFM structure was analyzed in heterozygous mutants in wild type and *dDAAM^Ex1^* mutant backgrounds. The results revealed that the mild *dDAAM^Ex1^* IFM phenotype (Figure 6A) is strongly enhanced by *Act88F^KM88^* and enhanced by *Tm2^3^*. Myofibrils of *Act88F^KM88^* heterozygotes were thinner than wild type and some Z-discs were not entirely straight (Figure 6B), but the precisely repeating organization of the sarcomeres remained. In contrast, the IFM of *dDAAM^Ex1^*; *Act88F^KM88^*/+ double mutants exhibited a network of very thin myofibrils often with a branched appearance, in which Z-disc and sarcomeric organization appeared to be completely abolished (Figure 6C). Similarly, in *dDAAM^Ex1^*; *Tm2^3^*/+ mutants the myofibrils appeared disorganized, displaying strongly varying width, unequal sarcomere and thin filament length and the frequent appearance of mini-sarcomeres (Figure 6E). As controls we examined *Act5C* null mutants, affecting the major non-muscle cell specific actin isoform [31] and a strong loss-of-function allele of the cytoplasmic *Tm1* isoform, *Tm1^02299^* [32]. As expected, these mutations did not alter the IFM phenotype of *dDAAM^Ex1^* (Figure S5). The strong dominant genetic interaction between *dDAAM* and the IFM-specific *Act88F* and *Tm2* alleles, and the complete lack of interaction with the non-muscle cell specific isoforms, suggests that the major function of dDAAM during muscle development is indeed linked to the regulation of sarcomeric actin filament formation.

### dDAAM is required for thin filament elongation

Under *in vitro* conditions the FH2 or FH1–FH2 domains of dDAAM behave as *bona fide* formins possessing both actin nucleation and elongation activities [33]. The observation that the thin filaments are shorter in *dDAAM* mutants than in wild type, suggested that dDAAM is a positive regulator of thin filament elongation. Consistent with the view that muscle thin filaments elongate from their pointed ends, dDAAM is present at the pointed end of actin filaments in the IFM, although, as expected for a formin, it also accumulates at barbed ends. To determine whether dDAAM is functionally important for pointed end elongation we investigated genetic interactions of *dDAAM* with mutations affecting the pointed end regulator proteins SALS and Tmod. SALS promotes filament elongation *in vivo* [18], whereas Tmod binding is thought to prevent elongation [16]. The presence of *sals^f07849^*/+ in a *dDAAM^Ex1^* mutant background had no obvious phenotypic effect. In contrast, the *tmod^00848^* mutation entirely suppressed the weak flightless phenotype of *dDAAM^Ex1^* (4.9±0.5%, mean±SEM, n = 160, p = 0.027) (Figure 1A) suggesting that dDAAM and Tmod may act antagonistically during thin filament growth.

**Figure 7:**
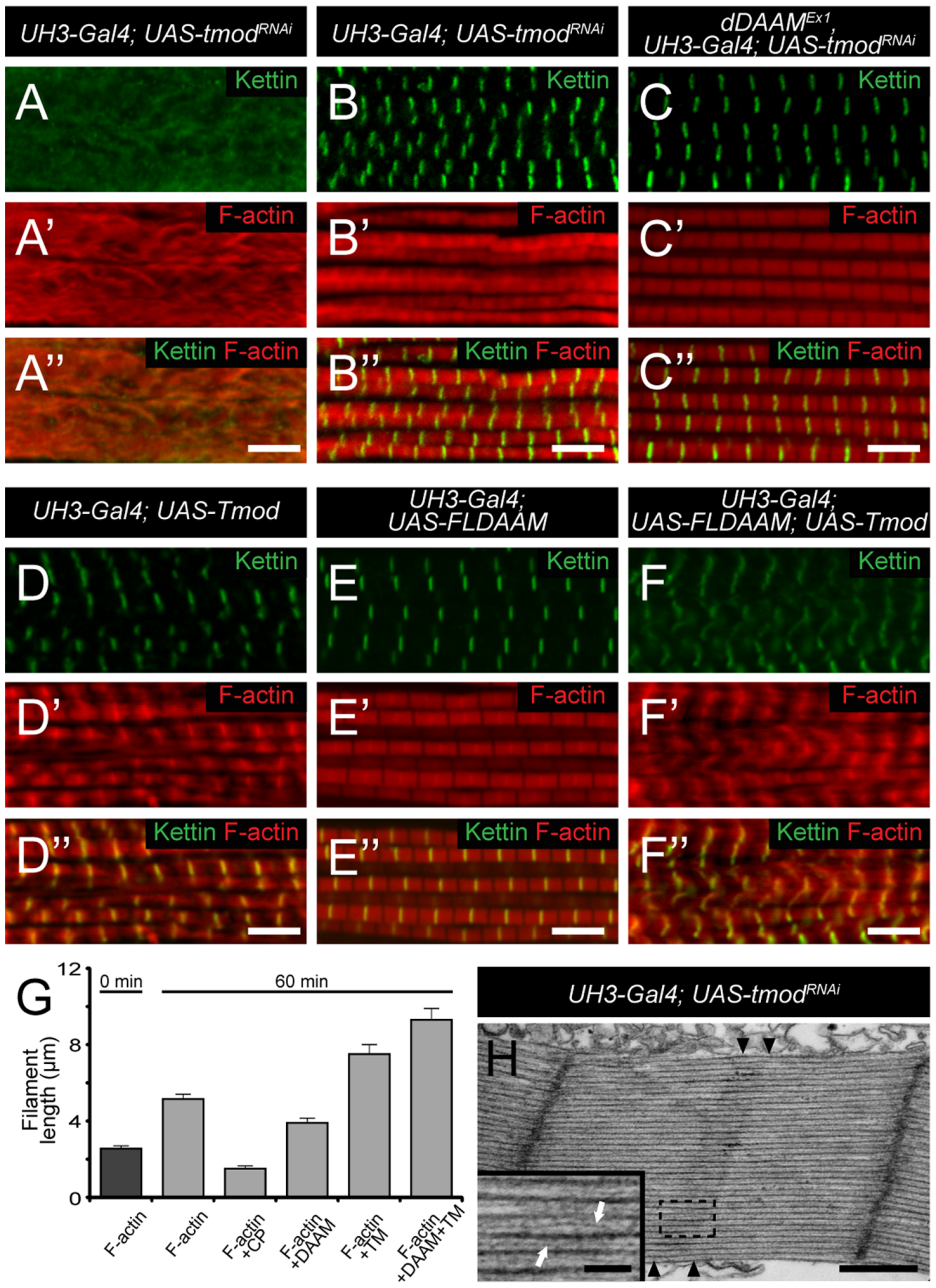
The interaction of *dDAAM* and *tmod*. Upon silencing of *tmod* myofibrils get severely disrupted (A–A″), though ∼10% of them show a milder effect with regular Z-disc arrangement but missing H-zones (B–B″). In *dDAAM^Ex1^*, *UH3-Gal4*; *UAS-tmod^RNAi^* muscles most myofibrils have a nearly wild type sarcomeric organization with regularly spaced Z-discs and M-lines, and almost normal sarcomere length (C–C″). (D–D″) In *UH3-Gal4*; *UAS-Tmod* IFMs the sarcomeric thin filaments often appear to be shorter than wild type as judged by phalloidin staining, whereas myofibrils of *UH3-Gal4*; *UAS-FLDAAM* muscle look wild type (E–E″). Simultaneous overexpression of FLDAAM and Tmod results in the same effect as the expression of Tmod alone (F–F″; compare to D–D″). Kettin in green, actin in red in A–F″. (G) An end-to-end actin annealing assay, dark grey: 0 minute control, average filament length in the presence of 1 µM F-actin (F-actin), light gray: average filament length after 60 minutes incubation, in the presence of 1 µM F-actin (F-actin), 1 µM F-actin+ 10 nM capping protein (F-actin+CP), 1 µM F-actin+100 nM DAAM-FH1-FH2 (F-actin+DAAM), 1 µM F-actin+1 µM skeletal tropomyosin (F-actin+TM), 1 µM F-actin+100 nM DAAM-FH1-FH2+1 µM skeletal tropomyosin (F-actin+DAAM+TM). Bars represent mean values with respective SEMs. (H) Electronmicrograph of a *tmod^RNAi^* IFM. Black arrowheads mark the borders of the mid-sarcomeric region where the M-line structures are not evident but thin filaments appear to cross this area. White arrows on the inset, corresponding to the dashed area, mark thin filaments that fail to terminate in the H-zone. Bars: 5 µm (A–F″); 500 nm (H) 100 nm (H, inset).

To investigate the dDAAM/Tmod interaction in more detail we first examined the IFM-specific RNAi silencing of *tmod*, and we found that in most myofibrils it severely disrupted myofibrillogenesis (Figure 7A). However, approximately 10% of the myofibrils had almost normal looking Z-discs allowing us to determine that these sarcomeres were shorter (2.62±0.11 µm; n = 26; mean±SD; p<0.001) than wild type. Phalloidin staining revealed the presence of thin filaments in the mid-sarcomeric region (Figure 7B) and impaired M-lines are evident by EM analysis (Figure 7H). The strong effect on myofibrillogenesis is in accordance with previous reports that *Tmod1* in mouse and *Unc-94* (*tmd-1*) in *C. elegans* are required for myofibril assembly [34], [35], [36], [37]. The decreased sarcomere length was unexpected as the inhibition of *Tmod* function increases sarcomere length in cultured cardiomyocytes [38] or in *Drosophila* primary muscles [18]. We noted however, that although sarcomere length of *UH3-Gal4; UAS-tmod^RNAi^* flight muscles was reduced, some of the thin filaments clearly failed to terminate in the H-zone of these mutant sarcomeres (Figure 7H). Therefore, individual filament length can be longer than in wild type, which would be consistent with the known function of Tmod in filament length regulation. To study whether the *tmod^RNAi^* phenotype is sensitive to dDAAM protein level, *tmod* silencing was carried out in a *dDAAM^Ex1^* mutant background. Most (∼80%) myofibrils displayed a striated pattern with distinct M-lines and somewhat aberrant Z-discs, and nearly normal sarcomere length (2.8±0.13 µm; n = 30; mean±SD; p<0.001) (Figure 7C). This phenotype suggests that the reduced dDAAM levels suppress the “over elongation” of the thin filaments seen in the IFM of *tmod^RNAi^* flies, and hence, these results further confirm that these two proteins have antagonistic activities in thin filament elongation.

Although dDAAM protein is detected in the vicinity of the pointed ends of sarcomeric thin filaments, former structural studies indicated that formins are strictly barbed end binding proteins [21], [39], [40]. This paradox would be resolved if pointed end elongation relies on the formation of short actin filaments that anneal sequentially to growing thin filaments anchored to the Z-disc. In this model, dDAAM would mediate the assembly of short actin filaments by acting as a classical barbed end binding formin, but would additionally either actively promote actin filament annealing, or at least not block it. To test this expectation, an *in vitro* F-actin annealing assay was carried out with the barbed end binding FH1–FH2 domains of dDAAM. We found that the presence of the FH1–FH2 fragment (100 nM) allowed the end-to-end annealing of actin filaments (Figure 7G), although in previous *in vitro* assays the FH1–FH2 domains of dDAAM significantly reduced barbed end assembly under similar conditions [33]. Capping protein and TM were used as controls. In accordance with former studies [41], [42], the barbed end blocking capping protein had an inhibitory effect, whereas TM enhanced the end-to-end annealing of actin filaments, and the combined effect of TM and dDAAM was even slightly higher than the one of TM alone (Figure 7G). The annealing model suggests that, even if at the pointed end sarcomeric region, dDAAM acts as a barbed end binding protein. Hence it follows that dDAAM is unlikely to directly interfere with the binding of pointed end cappers, such as Tmod. To address this issue, we investigated the effect of dDAAM and Tmod in overexpression assays. The IFM specific overexpression of Tmod resulted in thin filament shortening [43] (Figure 7D–D″), whereas the excess of dDAAM had no obvious phenotypic effect in the IFM (Figure 7E–E″). When the two proteins were expressed together, we observed the same phenotypic effect as the overexpression of Tmod alone (Figure 7F–F″). Therefore these results support the annealing model of dDAAM mediated thin filament elongation and the interaction studies are also consistent with the proposal that dDAAM affects thin filament assembly at pointed ends.

## Discussion

The sarcomeric actin filaments are critical structural and functional elements of muscles, yet the mechanism of actin filament formation and its regulation during myofibrillogenesis remained unclear. The initial steps of actin filament formation require nucleation factors, of which Lmod and Fhod3 have been previously identified as muscle-specific nucleators [6], [7]. However, functional analysis led to the conclusion that Lmod and Fhod3 are crucial to myofibril maintenance but are unlikely to contribute to filament nucleation during the initial stages of myofibril assembly. Recent work in *C. elegans* revealed that two members of the formin family, Cyk-1 (a Diaphanous ortholog) and Fhod-1, are both enriched at the Z-disc and promote filament lattice growth and its maintenance in striated muscles [44]. Surprisingly however, the muscle phenotypes displayed by *cyk-1* or *fhod-1* single mutants were relatively mild and it is unresolved whether other nucleation factors are required in worm muscles. Here we provide *in vivo* evidence that DAAM, another formin family member, is important for sarcomeric thin filament formation. We have found that dDAAM is required for thin filament elongation and that the actin-assembling activity of dDAAM is indispensable for formation of functional muscles. In addition, we have shown that in differentiating C_2_C_12_ cells the mouse Daam1 ortholog is incorporated into sarcomeric complexes at least as early as α-actinin. Thus DAAM family formins are strong candidates for being involved in the initial assembly of myofibrillar actin filaments. Interestingly, although the F-actin content of *dDAAM* mutant muscles is reduced, some filaments still form. Notably however, the *dDAAM* mutants available for muscle studies are not protein null. This prevents us from determining whether an additional nucleation factor, such as Dia or Fhos, is involved or that residual dDAAM activity is sufficient to promote some level of F-actin formation. Nevertheless, our results demonstrate that *dDAAM* is a developmentally important sarcomere-associated actin assembly factor in *Drosophila*. Remarkably, expression of the vertebrate DAAM orthologs are known to be abundant in developing somites and heart [23], [45], and genetic analysis of *mDaam1* indicated a role in sarcomere organization in cardiomyocytes [23]. Overall this suggests that the regulation of sarcomeric actin filament formation is an evolutionary conserved DAAM function.

**Figure 8:**
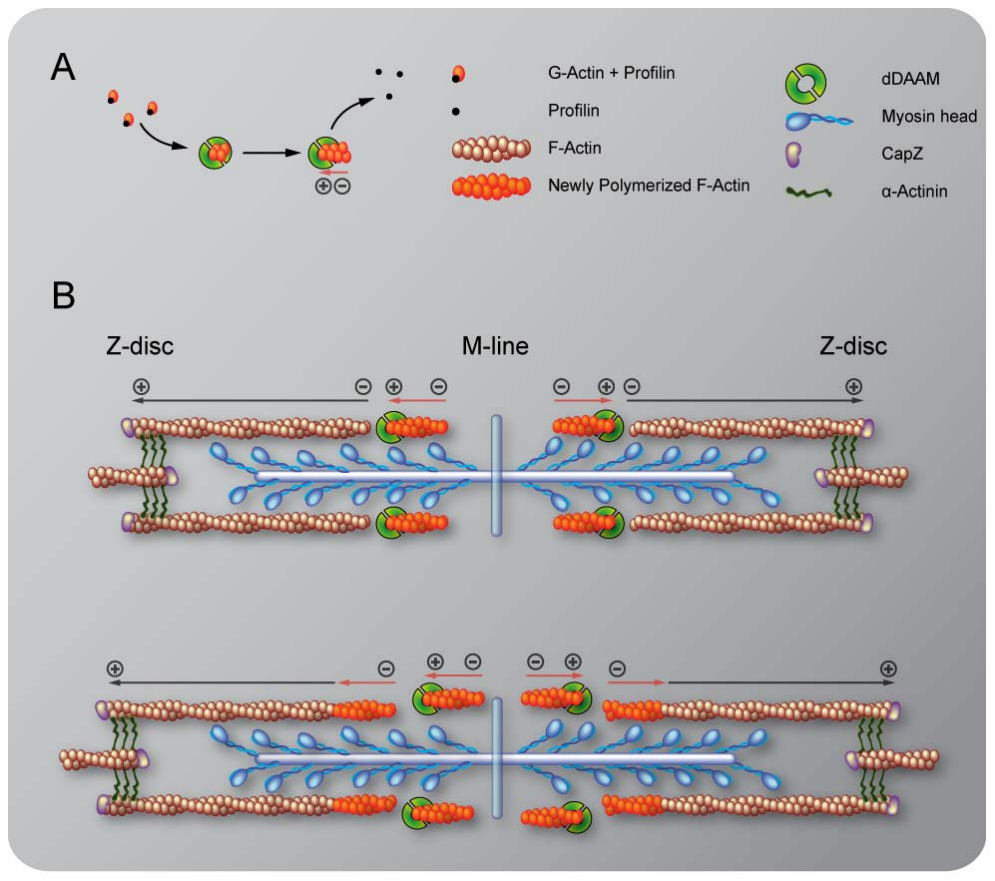
A model of DAAM mediated ‘pointed end elongation’. (A) Nucleation and elongation of short actin filaments by the barbed (+) end binding formin DAAM. (B) A possible mechanism of thin filament elongation from the pointed end (−) is the end-to-end annealing of DAAM assembled short actin filaments (in orange) to the Z-disc anchored growing “mother filament” (in brown).

Our studies revealed that in the IFM the dDAAM protein is mostly enriched at either end of the thin filaments, the expected positions for proteins affecting thin filament assembly. We formerly showed that *in vitro* dDAAM behaves as a *bona fide* formin, possessing all the major properties reported for other formin family members [33]. Here we propose that at Z-discs dDAAM may regulate G-actin incorporation with the well described barbed end processive capping mechanism of formins. Given that the sarcomeric dDAAM expression in the IFM, including the Z-disc accumulation, is maintained during adulthood, it appears likely that dDAAM also contributes to the maintenance of normal muscle structure and function. Besides the Z-disc enrichment, dDAAM also accumulates at the pointed end region of the thin filaments. Since dDAAM promotes thin filament formation and acts antagonistically to the F-actin pointed end capping protein, Tmod, the simplest interpretation of these data is to assume that dDAAM is involved in filament elongation from the pointed end. This is in good accordance with the evidence that in cardiac myocytes and in *Drosophila* primary cultures actin dynamics predominate at the pointed ends [17], [18], yet the presence at the pointed ends is unexpected for a formin, a barbed end binding protein. Because available structural studies exclude the possibility that a formin directly binds to the pointed end, dDAAM might be recruited to the pointed end by binding to a different protein than actin, or our findings indicate the presence of F-actin barbed ends in the vicinity of the pointed end of the thin filaments. Although we cannot strictly exclude the first possibility, at present the functional importance of such an association is unclear. Therefore we favor the second alternative that has interesting mechanistic implications. If barbed ends indeed exist in the region of the pointed ends, then pointed end elongation could be achieved through the end-to-end annealing of short actin filaments to the Z-disc anchored growing “mother filament” (Figure 8). Such a mechanism, demonstrated *in vitro*, would allow rapid filament elongation at the pointed ends. Our data are compatible with the model in which dDAAM promotes the formation of these short filaments by acting as an F-actin barbed end binding processive capper that also allows filament annealing. An important question is how long these short filaments are? In this regard, it is interesting to note that during contractile ring formation in fission yeast the formin Cdc12p was shown to nucleate short actin filaments that anneal to each other in the presence of TM [42], and consistently, TM increased the annealing process by ∼2 fold in our *in vitro* assay. As TM is a major myofibrillar protein, and the IFM-specific *Tm2* mutation dominantly enhanced the thin filament defects of *dDAAM^Ex1^*, we propose that the length of the filaments involved in the annealing process is unlikely to be shorter, but could be equal to an F-actin fragment covered by one TM dimer which is about 37–38 nm or 14 actin monomers. Whereas the ability to anneal end-to-end is an intrinsic property of actin filaments, a better understanding of this mechanism during myofibril formation awaits future studies, most importantly the visualization of the short protofilaments. Nonetheless, it is remarkable that the formin Fhod3, implicated in myofibril maintenance and maturation [10], [46], also displays an accumulation in the pointed end region [7], [47] and might regulate actin assembly with a similar mechanism as dDAAM.

Previously presented models of thin filament growth in Drosophila proposed a two-step mechanism [18], [43]. According to this view, during the first step short filaments are assembled, and during the second step these filaments extend to their final length. Moreover, it is presumed that, at least in larval muscles, the initial phase is SALS-independent, whereas subsequent elongation from the pointed end requires SALS activity that is thought to antagonize the effect of Tmod [18]. The shorter sarcomeres observed in *dDAAM* mutant muscles argue that dDAAM is required during the second step of thin filament formation. On the other hand, the severe Z-disc organization defects and the reduced sarcomere number in larval muscles, that are also typical for *dDAAM* mutants, indicate an earlier function that may be linked directly to the initial steps of thin filament formation. Our mDaam1 protein localization data during C_2_C_12_ cell differentiation is also consistent with an early function during sarcomerogenesis, therefore dDAAM is a good candidate for being involved already in the first steps of sarcomeric thin filament formation. Whether the annealing mechanism is at work during the first, second or both steps of actin filament formation/elongation, and whether SALS and dDAAM cooperate or act through independent mechanisms during the second step, remain open questions.

Interestingly, beyond the strong effect on thin filaments, *dDAAM* also affects thick filament and myofibrillar lattice organization. While these phenotypes can be the indirect consequences of the severe impairment of the sarcomeric thin filament system, another alternative could be that *dDAAM* plays a more complex role in sarcomerogenesis. In favor of this idea we note that the *dDAAM* mutants display a more poorly organized filament system than observed in *Act88F* null mutants which completely lack the sarcomeric thin filaments [48]. Additionally, *dDAAM* affects the shape of the thick filaments which is not reported for *Act88F* [48]. Moreover, we found that despite the lack of thin filaments, in *Act88F* mutants the dDAAM protein remains associated with the muscle fibers displaying a non-uniform distribution with foci that largely overlap with those of Myosin staining (Figure S4C). Taking all these observations together with the unusually strong effect on lattice organization, we speculate that, besides actin binding, dDAAM might play an important role in the integration of the thin and thick filament systems during sarcomerogenesis. Remarkably, unlike the actin isoforms [49], overexpression of the wild type dDAAM protein in larval muscles significantly increased sarcomere number and muscle size while sarcomere length remained nearly normal. Therefore dDAAM appears to play an instructive role in sarcomere formation, and to our knowledge, this is the first example reported where overexpression of a single muscle protein results in such an effect on muscle development.

## Materials and Methods

### Fly strains and genetics

Unless indicated otherwise, flies were raised and crossed at 25°C according to standard procedures. *w^1118^* was used as wild-type control. In addition, the following fly stocks were used: *dDAAM^Ex1^*, *dDAAM^Ex68^*/*FM7c, Kr-GFP* and *w; UAS-FLDAAM* or *UAS-DAAM-PB* [50], *y w; DMef2-Gal4* (Bloomington Stock Center), *w; UH3-Gal4* [19], *w; UAS-Dcr2* (Bloomington), *ry^506^ tmod^00848^/TM3* (Bloomington), *w; UAS Tmod* (gift from J. Bai, Harvard Medical School, Boston), *ry^506^ Act88F^KM88^ e* [51], *y w; Tm2^3^* (Bloomington), *ry^506^ Tm1^02299^/TM3* (Bloomington), *w; sals^f07849^/TM6B* (Bloomington), *w Act5C^G0025^/FM7c* (Bloomington), *w; sls-GFP* [52], *w; UAS-Tmod^RNAi^* (NIG-FLY, Kyoto) and *w; UAS-dDAAM^KK102786^* (VDRC, Vienna).

The *UAS-dDAAM^RNAi-T129M^ dDAAM* specific RNAi line, targeting nucleotides 2562–3068 of the RE67944 dDAAM cDNA clone, was created by standard cloning and transformation techniques. To create a UAS-DAAM-PD clone, the PD isoform specific region was amplified from a cDNA pool generated by reverse transcription of mRNAs isolated from the L3 stage. We first created a pENTR3C-DAAM-PD clone that subsequently was used to create pTW-DAAM-PD (UAS-DAAM-PD) destination clones suitable for transgenesis.

The *UAS-DAAM-PB^I732A^* and *UAS-DAAM-PD^I1042A^* mutants were created by standard cloning techniques using pENTR3C-DAAM-PB and pENTR3C-DAAM-PD as templates for *in vitro* mutagenesis.

The *dDAAM^EGFP^* knock-in mutant was created by a two-step P-element mediated gene conversion experiment. First a targeting construct was assembled in a modified pBS vector where we inserted a 1.3 kb 3′ *dDAAM* genomic region until the last codon, this was followed by a 2.3 kb Gal4::VP16 fragment flanked with I-SceI cut sites on both sides, next we inserted a 1150 bp fusion fragment containing the 3′ *dDAAM* region encoding the last 83 C-terminal aminoacids fused to an EGFP coding sequence ending with a stop codon. This was followed with the entire 3′ UTR of dDAAM and a 1.1 kb genomic region further downstream of it. This way, besides the genomic flanking sequences, the construct carries Gal4::VP16 that can be used as a marker gene which is flanked both by I-SceI sites and a ∼250 bp long genomic duplication encoding the most C-terminal dDAAM coding sequences. This targeting construct was converted into the *dDAAM* genomic region after remobilizing the *EP(1)1542* P-element insertion located 200 bp downstream of dDAAM (see Flybase for details). To this end, *EP(1)1542* virgins were crossed to *ry^502^ Fab-7^1^ Δ2-3* (gift from L. Sipos, BRC HAS, Szeged) males and the embryonic progeny of this cross was injected with the targeting construct. Offspring of the previous cross was crossed to *w; UAS-EGFP* flies *en masse* and put on egg laying medium. Embryos were collected on apple-juice plates, and the hatching larvae were screened for GFP fluorescence with an MZ FLIII stereo microscope (Leica, Switzerland). Larvae with GFP expression in the tracheal and nervous system were collected individually and used to set up stocks. Conversion events were confirmed by PCR and sequencing. Once the Gal4::VP16 containing construct has been successfully converted into the *dDAAM* gene, in a subsequent round of crosses I-SceI was used to induce DNA double strand breaks that could eventually be repaired through homologous recombination between the ∼250 bp duplicated dDAAM regions. This event, confirmed by PCR and sequencing, led to the removal of all non *Drosophila* sequences with the exception of EGFP, and resulted in a C-terminally GFP-tagged *dDAAM* allele. *dDAAM^EGFP^* is fully viable and fertile in hemi- or homozygous state indicating that the presence of EGFP does not significantly alter dDAAM function.

### Immunohistochemistry

Newly eclosed adult IFMs were dissected from bisected half thoraces in 4% paraformaldehyde (PF), incubated for 15 minutes, then washed with relaxing solution (6 mM MgCl_2_, 5 mM EGTA, 5 mM ATP, 90 mM potassium propionate, 20 mM NaPi, pH 7.0). The muscles were permeabilized overnight in Triton-X/glycerol solution (50% v/v glycerol, 0.5% Triton X-100, 20 mM NaPi, 2 mM MgCl_2_, 1 mM EGTA, 5 mM DTT, pH 7.0) at 4°C, and washed in PBS supplemented with 0.5% Triton X-100 (PBT), then labeled with primary and secondary antibodies. For pupal IFM preparations, timed pupae were removed from their puparia and pinned by the head on dry Sylgard (Dow Corning), dorsal side down, and then submerged into 4% PF in PBS. After dissection along the ventral midline, unattached material was flushed gently away using a syringe to expose the IFMs. These were detached and incubated in fixative (4% PF in PBS) for a further 15 minutes, then transferred back to relaxing solution. For larval heart tube dissections larvae were cut along the ventral midline in relaxing solution. Then fixed with 4% PF in PBS [53]. Fat bodies and other organs were removed, then labeled with primary and secondary antibodies. For developmental staging, white pre-pupae with everted spiracles were removed into fresh vials at 25°C and harvested at required time-points. Adult flies were selected as ‘newly eclosed’ between 0 and 8 hr post-eclosion.

Primary antibodies, listed below, were applied overnight at 4°C. Muscles and heart tubes were washed three times in PBST, secondary antibodies were applied for 3 hr, then samples were rinsed three times again in PBST. The following primary antibodies were used: rat monoclonal anti-Kettin (MAC 155, 1∶200; Abcam); rat monoclonal anti-Myosin (MAC 147, 1∶200; Abcam), rabbit polyclonal anti-GFP (1∶1000; Sigma) and rabbit polyclonal anti-dDAAM (1∶1000) [22]. For secondary antibodies we used the appropriate Alexa-488, Alexa-546 and Alexa-647 (1∶600), actin was stained with Rhodamine-Phalloidin (1∶100) (all from Life Technologies). Samples were mounted in PBS∶glycerol (1∶4). Confocal images were captured on an Olympus FV1000 LSM microscope, images were edited with ImageJ (NIH) and Olympus FW10-ASW (version1.7a.).

### Larval length measurements and crawling assays

Aged larvae were collected and rinsed with tap water, and then gently placed onto agar plates. The plates were placed under an Olympus SZX12 dissecting microscope equipped with an Olympus C7070 digital camera. Total illumination was applied, images were acquired at 25 Hz. The recording environment (temperature, humidity, illumination) was stationary. Twenty seconds of movie was recorded with DScaler (The DScaler Project Team) for each larva. During this period of time most wild-type larvae moved out from the field of view. ImageJ (NIH) software was used to analyze the image sequences. Persistent forward movements were selected to characterize larval crawling velocity and larval length. Larval length was calculated as the average of the minimum and the maximum head to tail distance for each larva. Maximum intensity projections were used to generate larval tracks. To calculate larval crawling velocity, the lengths of these tracks were divided by the time. Tracked larvae were dissected, stained and subjected to muscle measurements made on VL3 (ventral longitudinal 3) muscles. Muscle length was measured manually as the major axis of the VL3 muscles; muscle width was measured as the minor axis of the VL3 muscles. Sarcomere number and sarcomere length were measured from gray scale intensity plots across phalloidin stained sarcomeres, sarcomere size being the distance between adjacent peaks.

### Flight tests

Flight tests were carried out with three day old flies [54]. Flies were released inside a perspex box illuminated from above, and scored for the ability to fly up, horizontally or down. Flies falling into the third category (down) were counted as flightless.

### Tissue culture and mouse muscles

The mouse myogenic cell line, C_2_C_12_ (ATCC), was maintained in growth medium (DMEM supplemented with 10% FBS; GIBCO/Life Technologies). Cells were initially plated into 100-mm-diameter dishes (Greiner) at a density of 10^4^/cm^2^. When cultures reached ∼80% confluence they were subcultured onto sterile glass coverslips in 35-mm-diameter dishes. Cultures were kept in growth medium until they reached 60% confluence and subsequently were switched to differentiation medium (DMEM containing 2% horse serum; GIBCO/Life Technologies). This medium was replaced every day, and samples were processed for immunostaining at selected time points. Cells were fixed in 4% formaldehyde in PBS for 10 minutes, and permeabilized in PBS+0.1% Triton-X100 for 3 minutes before staining. Primary antibodies were applied for 1 hr RT, and after 3×5 minutes washing in PBS, cells were incubated with secondary antibodies for another 1 hr. After washing three times for 5 minutes in PBS, samples were mounted in PBS∶glycerin (1∶4).

For sections of *m. tibialis anterior* and *m. vastus lateralis*, C57Bl/6 adult male mice were sacrificed by cervical dislocation. Leg muscle was dissected, embedded in Tissue-Tek O.C.T. compound (Sakura Finetek) and snap-frozen in isopentane cooled by liquid nitrogen. 10 µm cryosections were fixed in prechilled acetone and kept at −80°C.

For mammalian muscle and C_2_C_12_ staining the following antibodies were used: rabbit polyclonal anti-mDaam1 (1∶2000; Sigma), rabbit polyclonal anti-mDaam1 (1∶200; Abnova), mouse monoclonal anti-α-actinin (1∶80; Sigma), mouse monoclonal anti-titin 9D10 (1∶20; DSHB) and mouse monoclonal anti-myomesin (B4, 1∶1; DSHB). For secondary antibodies we used the appropriate Alexa-488, Alexa-546 and Alexa-647 (1∶600; Life Technologies). Images were taken and analyzed in a similar ways as flight muscles described above.

### Electron microscopy

Muscles were dissected and fixed in 3.2% paraformaldehyde, 0.5% glutaraldehyde, 1% sucrose, 0.028% CaCl_2_ in 0.1 N sodium cacodylate (pH 7.4) overnight at 4°C, and washed 2× overnight in 0.1 N sodium cacodylate (pH 7.4) at 4°C. Samples were postfixed in 0.5% osmium-tetroxide for 1 hr at room temperature, and embedded into Durcupan (Fluka) by following the manufacturer's recommendations. 70–80 nm ultrathin sections were prepared from 2–3 animals per genotype, stained in Reynold's lead citrate, and evaluated using a JEM-1011 electron microscope (JEOL) equipped with Morada camera and iTEM software (Olympus).

### AFM and force measurements

IFM muscle fibers, falling apart for individual myofibrils upon preparation, were mounted on a poly-L-lysine coated glass surface and measured in phosphate buffered saline. To identify the target points at which to perform individual force measurements, a rough and low resolution scan was taken (not shown). Experiments were carried out with Asylum MFP-3D head and controller (Asylum Research, Santa Barbara, CA). The driver program was written in IGOR Pro software (version 5.04, Wavemetrics, Lake Oswego, OR). Rectangular, gold coated, silicon nitride cantilevers were used, with a nominal spring constant of 30 pN/nm and a V shaped tip with radius of curvature of roughly 30 nm (Bio-Lever, BL-RC150 VB-C1, Olympus Optical Co. Ltd). The measurements were performed in contact mode in liquid, with the vertical piezo working in a closed loop. Constant speed of 0.6 µm/s (scan rate 0.1 Hz) and total load was kept below 1 nN during experiments.

Simple force curves were measured by lowering the probe until a desired deflection is reached and pulling it back. To calculate the sample's elasticity the contact region of the lowering part from force curves has been used. By subtracting a reference curve, recorded on a hard surface from those measured on the object of interest, the sample's force vs. indentation curve can be obtained, which provides the Young's modulus of the measured sample [55]. Several points were examined recording multiple force curves at each selected place; the average and standard deviation of which was calculated.

### Protein gel electrophoresis and western blot analysis

Adult IFM samples were dissected as described above. Tissues were immediately placed in ice-cold RIPA lysis buffer and kept overnight. SDS-PAGE and Western blot analyses were carried out according to standard protocols. Membranes were stained with rabbit anti-dDaam (1∶1000), and rabbit anti-glycogen phosphorylase (1∶20000) (gift from A. Udvardy, BRC HAS, Szeged) used as a loading control. Secondary antibody was α-rabbit-HRPO (1∶10000; Sigma). For chemiluminescent detection we used a Millipore Immobilon kit.

### Actin filament annealing tests

To measure the annealing of actin filaments fluorescence microscopy assays were performed. Actin filaments (10 µM, F-actin) were polymerized for 2 hr at room temperature in 4 mM Tris-HCl (pH 7.0), 0.1 mM CaCl2, 0.2 mM ATP, 0.5 mM DTT, 1 mM EGTA, 1 mM MgCl2 and 50 mM KCl (F-buffer). The F-actin solution was then diluted to 1 µM using F-buffer in the absence or presence of actin-binding proteins (100 nM capping protein or 100 nM dDAAM FH1-FH2 or 1 µM skeletal muscle TM or 100 nM dDAAM FH1-FH2 and 1 µM skeletal muscle TM). The samples were incubated overnight. For investigation of the annealing, Alexa-488-phalloidin labeled samples were sheared five times through a 26 gauge needle. Samples were diluted 100 fold into microscopy buffer (F-buffer supplemented with 50 mMDTT, 5 mM DABCO and 0.5% (w/v) methylcellulose) 0 and 60 minutes after shearing and processed for microscopy observations. Single actin filaments were observed with an Olympus IX81 inverted fluorescence microscope using a 100× objective (NA1.4) and a CCD camera (Orca ERG Hamamatsu). The length of the actin filaments was measured and analyzed with ImageJ. Under each condition 3–4 independent measurements were performed and 300–600 filaments were analyzed. Statistical analysis was carried out using Microsoft Excel or Microcal Origin 6.0.

### Statistics

Excel (Microsoft) was used to collect and organise data. Statistical analysis was carried out using Prism 5 (GraphPad Software Inc.) and/or SigmaPlot 12 (Systat Software Inc.). Normality of the data was verified by Shapiro-Wilk test. Pairwise comparisons were made using the Student's *t* test or the Mann-Whitney *U* test according to the normality, p<0.05 was considered as statistically significant.

## Supporting Information

Figure S1 Impaired adult IFM structure in *dDAAM* mutants. (A–A″) IFM myofibrils of a flightless *dDAAM^Ex1^* mutant looks largely normal, although some of the sarcomeres show reduced lengths (2.5 µm instead of 3.2 µm; Kettin in green, actin in red). (B–C″) Myofibrils of wild type (B–B″) and *dDAAM^Ex1^*, *UDT* mutants (C–C″) stained for Myosin (green) and actin (red). Note the severely impaired Myosin and M-line organization, and the strong reduction of F-actin level in IFM of the *dDAAM* mutant (C). In newly eclosed (D, D′) and 4 day-old (E, E′) *dDAAM^EGFP^* adults anti-GFP staining is evident at the Z-disc (arrows) and M-band (arrowhead). (F) Coomassie staining shows no significant difference in the amount of G-actin in wild type and *dDAAM^Ex1^*, *UDT* mutants. Bars, 5 µm.
(TIF)

Figure S2 *dDAAM* impairs pupal IFM structure and the mechanical properties of muscles. Myofibrils from a wild type (A–A″) and *dDAAM^Ex1^*, *UDT* mutant (B–B″) pupal IFM (48 hours APF, 29°C) stained for actin (in red) and Kettin (in green). The mutant IFM shows Z-disc and M-line organization defects. (C) Quantification of the transverse elasticity of wild type and *dDAAM* mutant myofibrils measured by Atomic Force Microscopy. To characterize the mechanical properties of the myofibrils, their transverse elasticity (Young's modulus) was calculated. The average curve is fitted with a second order polynomial (C). The elasticity of *dDAAM^Ex1^* and *dDAAM^Ex1^*, *UDT* (RNAi) mutant fibers is significantly lower, 6±1.63 kPa (n = 35) and 4±1.24 kPa (n = 15), respectively, than the one of wild type, 22±4.91 kPa (n = 25). Bars, 2 µm.
(TIF)

Figure S3 *dDAAM* affects IFM fiber morphology and heart tube development. (A–B) IFM structure of a wild type (A) and *dDAAM^Ex1^*, *UDT* mutant (B) as seen under confocal microscope. In these sagittal sections of thoraces rhodamine-phalloidin was used to visualize the muscle F-actin. Note that mutant dorsolongitudinal muscle (DLM) fibers are shorter (arrows) and thinner than in wild type, and some of the muscles appear degenerated. (C–D) Phalloidin staining of a wild type (C) and *dDAAM^Ex68^* mutant (D) larval heart tube to visualize F-actin (in green). Compared to wild type, the *dDAAM^Ex68^* mutant has reduced F-actin levels, and heart tube diameter is smaller. In addition, many of the mutant myofibrils appear thinner than their wild type counterparts and often deviate from the typical wild type orientation. (E–F) A developing wild type (E) larval body wall muscle at 72 hours AEL clearly expresses and accumulates the dDAAM protein (in cyan) in its myofibrils. A similar but weaker dDAAM expression pattern can still be detected in ∼10% of *dDAAM^Ex68^* mutant larvae (F) even at 100 hours AEL. Kettin (in green) labels the Z-discs in E-F. Bars: 100 µm (A–B); 40 µm (C–D); 5 µm (E–F).
(TIF)

Figure S4 Sarcomeric localization of the dDAAM protein in wild type and mutant IFMs. Myofibrils of wild type (A–A″), *dDAAM^Ex1^*, *UDT* (B–B″) and *Act88F* null mutant (C–C″) IFM from young adults stained for dDAAM (cyan, A–C″), actin (red, A′–B″) and Myosin (green, C′ and C″). Staining of wild type IFM reveals dDAAM accumulation at M-line and Z-disc, and in the sarcoplasm (A–A″). In contrast, in a *dDAAM^Ex1^*, *UDT* mutant IFM only a weak background staining is evident (compare A to B). In *Act88F* null mutants, which completely lack sarcomeric thin filaments, dDAAM protein remains associated with muscle fibers and displays a partial colocalization with myosin (C–C″). Bars, 5 µm.
(TIF)

Figure S5 *dDAAM* shows no interaction with the non-muscle cell specific isoforms of actin and tropomyosin. Adult IFM myofibrils showing *dDAAM^Ex1^* (A), *Act5C^G0025^*/+ (B), *dDAAM^Ex1^*; *Act5C^G0025^*/+ (C), *Tm1^02299^*/+ (D) and *dDAAM^Ex1^*; *Tm1^02299^*/+ (E) mutants stained for Kettin (green) and actin (red). Note that all mutant myofibrils look nearly wild type. Bars, 5 µm.
(TIF)

## References

[1] Luther PK (2009) The vertebrate muscle Z-disc: sarcomere anchor for structure and signalling. J Muscle Res Cell Motil 30: 171–185. 19830582 10.1007/s10974-009-9189-6 PMC2799012

[2] Sparrow JC, Schock F (2009) The initial steps of myofibril assembly: integrins pave the way. Nat Rev Mol Cell Biol 10: 293–298. 19190670 10.1038/nrm2634

[3] Pollard TD, Blanchoin L, Mullins RD (2000) Molecular mechanisms controlling actin filament dynamics in nonmuscle cells. Annu Rev Biophys Biomol Struct 29: 545–576. 10940259 10.1146/annurev.biophys.29.1.545

[4] Campellone KG, Welch MD (2010) A nucleator arms race: cellular control of actin assembly. Nat Rev Mol Cell Biol 11: 237–251. 20237478 10.1038/nrm2867 PMC2929822

[5] Chesarone M, Gould CJ, Moseley JB, Goode BL (2009) Displacement of formins from growing barbed ends by bud14 is critical for actin cable architecture and function. Dev Cell 16: 292–302. 19217430 10.1016/j.devcel.2008.12.001 PMC2667650

[6] Chereau D, Boczkowska M, Skwarek-Maruszewska A, Fujiwara I, Hayes DB, et al (2008) Leiomodin is an actin filament nucleator in muscle cells. Science 320: 239–243. 18403713 10.1126/science.1155313 PMC2845909

[7] Taniguchi K, Takeya R, Suetsugu S, Kan OM, Narusawa M, et al (2009) Mammalian formin fhod3 regulates actin assembly and sarcomere organization in striated muscles. J Biol Chem 284: 29873–29881. 19706596 10.1074/jbc.M109.059303 PMC2785617

[8] Skwarek-Maruszewska A, Boczkowska M, Zajac AL, Kremneva E, Svitkina T, et al (2010) Different localizations and cellular behaviors of leiomodin and tropomodulin in mature cardiomyocyte sarcomeres. Mol Biol Cell 21: 3352–3361. 20685966 10.1091/mbc.E10-02-0109 PMC2947471

[9] Tsukada T, Pappas CT, Moroz N, Antin PB, Kostyukova AS, et al (2010) Leiomodin-2 is an antagonist of tropomodulin-1 at the pointed end of the thin filaments in cardiac muscle. J Cell Sci 123: 3136–3145. 20736303 10.1242/jcs.071837 PMC2931607

[10] Iskratsch T, Lange S, Dwyer J, Kho AL, dos Remedios C, et al (2010) Formin follows function: a muscle-specific isoform of FHOD3 is regulated by CK2 phosphorylation and promotes myofibril maintenance. Journal of Cell Biology 191: 1159–1172. 21149568 10.1083/jcb.201005060 PMC3002041

[11] Iskratsch T, Reijntjes S, Dwyer J, Toselli P, Degano IR, et al (2013) Two distinct phosphorylation events govern the function of muscle FHOD3. Cell Mol Life Sci 70: 893–908. 23052206 10.1007/s00018-012-1154-7 PMC3696992

[12] Anhezini L, Saita AP, Costa MS, Ramos RG, Simon CR (2012) Fhos encodes a Drosophila Formin-like protein participating in autophagic programmed cell death. Genesis 50: 672–684. 22422652 10.1002/dvg.22025

[13] Castrillon DH, Wasserman SA (1994) Diaphanous is required for cytokinesis in Drosophila and shares domains of similarity with the products of the limb deformity gene. Development 120: 3367–3377. 7821209 10.1242/dev.120.12.3367

[14] Emmons S, Phan H, Calley J, Chen W, James B, et al (1995) Cappuccino, a Drosophila maternal effect gene required for polarity of the egg and embryo, is related to the vertebrate limb deformity locus. Genes Dev 9: 2482–2494. 7590229 10.1101/gad.9.20.2482

[15] Tanaka H, Takasu E, Aigaki T, Kato K, Hayashi S, et al (2004) Formin3 is required for assembly of the F-actin structure that mediates tracheal fusion in Drosophila. Dev Biol 274: 413–425. 15385168 10.1016/j.ydbio.2004.07.035

[16] Littlefield RS, Fowler VM (2008) Thin filament length regulation in striated muscle sarcomeres: pointed-end dynamics go beyond a nebulin ruler. Semin Cell Dev Biol 19: 511–519. 18793739 10.1016/j.semcdb.2008.08.009 PMC2650474

[17] Littlefield R, Almenar-Queralt A, Fowler VM (2001) Actin dynamics at pointed ends regulates thin filament length in striated muscle. Nat Cell Biol 3: 544–551. 11389438 10.1038/35078517

[18] Bai J, Hartwig JH, Perrimon N (2007) SALS, a WH2-domain-containing protein, promotes sarcomeric actin filament elongation from pointed ends during Drosophila muscle growth. Dev Cell 13: 828–842. 18061565 10.1016/j.devcel.2007.10.003

[19] Katzemich A, Kreiskother N, Alexandrovich A, Elliott C, Schock F, et al (2012) The function of the M-line protein obscurin in controlling the symmetry of the sarcomere in the flight muscle of Drosophila. J Cell Sci 125: 3367–3379. 22467859 10.1242/jcs.097345 PMC3516378

[20] Reedy MC, Beall C (1993) Ultrastructure of developing flight muscle in Drosophila. I. Assembly of myofibrils. Dev Biol 160: 443–465. 8253277 10.1006/dbio.1993.1320

[21] Xu Y, Moseley JB, Sagot I, Poy F, Pellman D, et al (2004) Crystal structures of a Formin Homology-2 domain reveal a tethered dimer architecture. Cell 116: 711–723. 15006353 10.1016/s0092-8674(04)00210-7

[22] Matusek T, Djiane A, Jankovics F, Brunner D, Mlodzik M, et al (2006) The Drosophila formin DAAM regulates the tracheal cuticle pattern through organizing the actin cytoskeleton. Development 133: 957–966. 16469972 10.1242/dev.02266

[23] Li DQ, Hallett MA, Zhu WQ, Rubart M, Liu Y, et al (2011) Dishevelled-associated activator of morphogenesis 1 (Daam1) is required for heart morphogenesis. Development 138: 303–315. 21177343 10.1242/dev.055566 PMC3005605

[24] Yaffe D, Saxel O (1977) Serial passaging and differentiation of myogenic cells isolated from dystrophic mouse muscle. Nature 270: 725–727. 563524 10.1038/270725a0

[25] Kontrogianni-Konstantopoulos A, Catino DH, Strong JC, Bloch RJ (2006) De novo myofibrillogenesis in C2C12 cells: evidence for the independent assembly of M bands and Z disks. Am J Physiol Cell Physiol 290: C626–637. 16207790 10.1152/ajpcell.00442.2005

[26] Trombitas K, Greaser M, French G, Granzier H (1998) PEVK extension of human soleus muscle titin revealed by immunolabeling with the anti-titin antibody 9D10. J Struct Biol 122: 188–196. 9724620 10.1006/jsbi.1998.3984

[27] Wang SM, Greaser ML, Schultz E, Bulinski JC, Lin JJ, et al (1988) Studies on cardiac myofibrillogenesis with antibodies to titin, actin, tropomyosin, and myosin. J Cell Biol 107: 1075–1083. 3047149 10.1083/jcb.107.3.1075 PMC2115289

[28] Grove BK, Kurer V, Lehner C, Doetschman TC, Perriard JC, et al (1984) A new 185,000-dalton skeletal muscle protein detected by monoclonal antibodies. J Cell Biol 98: 518–524. 6537951 10.1083/jcb.98.2.518 PMC2113097

[29] Karlik CC, Fyrberg EA (1985) An insertion within a variably spliced Drosophila tropomyosin gene blocks accumulation of only one encoded isoform. Cell 41: 57–66. 2986850 10.1016/0092-8674(85)90061-3

[30] Okamoto H, Hiromi Y, Ishikawa E, Yamada T, Isoda K, et al (1986) Molecular characterization of mutant actin genes which induce heat-shock proteins in Drosophila flight muscles. EMBO J 5: 589–596. 16453675 10.1002/j.1460-2075.1986.tb04251.x PMC1166803

[31] Fyrberg EA, Mahaffey JW, Bond BJ, Davidson N (1983) Transcripts of the six Drosophila actin genes accumulate in a stage- and tissue-specific manner. Cell 33: 115–123. 6432334 10.1016/0092-8674(83)90340-9

[32] Tetzlaff MT, Jackle H, Pankratz MJ (1996) Lack of Drosophila cytoskeletal tropomyosin affects head morphogenesis and the accumulation of oskar mRNA required for germ cell formation. EMBO J 15: 1247–1254. 8635457 PMC450027

[33] Barko S, Bugyi B, Carlier MF, Gombos R, Matusek T, et al (2010) Characterization of the biochemical properties and biological function of the formin homology domains of Drosophila DAAM. J Biol Chem 285: 13154–13169. 20177055 10.1074/jbc.M109.093914 PMC2857102

[34] Fritz-Six KL, Cox PR, Fischer RS, Xu B, Gregorio CC, et al (2003) Aberrant myofibril assembly in tropomodulin1 null mice leads to aborted heart development and embryonic lethality. J Cell Biol 163: 1033–1044. 14657235 10.1083/jcb.200308164 PMC2173615

[35] McKeown CR, Nowak RB, Moyer J, Sussman MA, Fowler VM (2008) Tropomodulin1 is required in the heart but not the yolk sac for mouse embryonic development. Circ Res 103: 1241–1248. 18927466 10.1161/CIRCRESAHA.108.178749 PMC2744601

[36] Stevenson TO, Mercer KB, Cox EA, Szewczyk NJ, Conley CA, et al (2007) unc-94 encodes a tropomodulin in Caenorhabditis elegans. J Mol Biol 374: 936–950. 17976644 10.1016/j.jmb.2007.10.005 PMC2175264

[37] Yamashiro S, Cox EA, Baillie DL, Hardin JD, Ono S (2008) Sarcomeric actin organization is synergistically promoted by tropomodulin, ADF/cofilin, AIP1 and profilin in C. elegans. J Cell Sci 121: 3867–3877. 18984629 10.1242/jcs.040477 PMC2615493

[38] Sussman MA, Baque S, Uhm CS, Daniels MP, Price RL, et al (1998) Altered expression of tropomodulin in cardiomyocytes disrupts the sarcomeric structure of myofibrils. Circ Res 82: 94–105. 9440708 10.1161/01.res.82.1.94

[39] Lu J, Meng W, Poy F, Maiti S, Goode BL, et al (2007) Structure of the FH2 domain of Daam1: implications for formin regulation of actin assembly. J Mol Biol 369: 1258–1269. 17482208 10.1016/j.jmb.2007.04.002 PMC1939941

[40] Shimada A, Nyitrai M, Vetter IR, Kuhlmann D, Bugyi B, et al (2004) The core FH2 domain of diaphanous-related formins is an elongated actin binding protein that inhibits polymerization. Mol Cell 13: 511–522. 14992721 10.1016/s1097-2765(04)00059-0

[41] Andrianantoandro E, Blanchoin L, Sept D, McCammon JA, Pollard TD (2001) Kinetic mechanism of end-to-end annealing of actin filaments. J Mol Biol 312: 721–730. 11575927 10.1006/jmbi.2001.5005

[42] Skau CT, Neidt EM, Kovar DR (2009) Role of tropomyosin in formin-mediated contractile ring assembly in fission yeast. Mol Biol Cell 20: 2160–2173. 19244341 10.1091/mbc.E08-12-1201 PMC2669024

[43] Mardahl-Dumesnil M, Fowler VM (2001) Thin filaments elongate from their pointed ends during myofibril assembly in Drosophila indirect flight muscle. J Cell Biol 155: 1043–1053. 11739412 10.1083/jcb.200108026 PMC2150893

[44] Mi-Mi L, Votra S, Kemphues K, Bretscher A, Pruyne D (2012) Z-line formins promote contractile lattice growth and maintenance in striated muscles of C. elegans. J Cell Biol 198: 87–102. 22753896 10.1083/jcb.201202053 PMC3392944

[45] Nakaya MA, Habas R, Biris K, Dunty WC Jr, Kato Y, et al (2004) Identification and comparative expression analyses of Daam genes in mouse and Xenopus. Gene Expr Patterns 5: 97–105. 15533824 10.1016/j.modgep.2004.06.001

[46] Kan OM, Takeya R, Abe T, Kitajima N, Nishida M, et al (2012) Mammalian formin Fhod3 plays an essential role in cardiogenesis by organizing myofibrillogenesis. Biol Open 1: 889–896. 23213483 10.1242/bio.20121370 PMC3507241

[47] Kan-o M, Takeya R, Taniguchi K, Tanoue Y, Tominaga R, et al (2012) Expression and subcellular localization of mammalian formin Fhod3 in the embryonic and adult heart. PLoS One 7: e34765. 22509354 10.1371/journal.pone.0034765 PMC3324543

[48] Beall CJ, Sepanski MA, Fyrberg EA (1989) Genetic dissection of Drosophila myofibril formation: effects of actin and myosin heavy chain null alleles. Genes Dev 3: 131–140. 2714648 10.1101/gad.3.2.131

[49] Roper K, Mao Y, Brown NH (2005) Contribution of sequence variation in Drosophila actins to their incorporation into actin-based structures in vivo. J Cell Sci 118: 3937–3948. 16105877 10.1242/jcs.02517

[50] Matusek T, Gombos R, Szecsenyi A, Sanchez-Soriano N, Czibula A, et al (2008) Formin proteins of the DAAM subfamily play a role during axon growth. J Neurosci 28: 13310–13319. 19052223 10.1523/JNEUROSCI.2727-08.2008 PMC6671601

[51] Mogami K, Hotta Y (1981) Isolation of Drosophila flightless mutants which affect myofibrillar proteins of indirect flight muscle. Mol Gen Genet 183: 409–417. 6801424 10.1007/BF00268758

[52] Morin X, Daneman R, Zavortink M, Chia W (2001) A protein trap strategy to detect GFP-tagged proteins expressed from their endogenous loci in Drosophila. Proc Natl Acad Sci U S A 98: 15050–15055. 11742088 10.1073/pnas.261408198 PMC64981

[53] Alayari NN, Vogler G, Taghli-Lamallem O, Ocorr K, Bodmer R, et al (2009) Fluorescent labeling of Drosophila heart structures. J Vis Exp (32) pii: 1423. 19826399 10.3791/1423 PMC3164059

[54] Cripps RM, Ball E, Stark M, Lawn A, Sparrow JC (1994) Recovery of dominant, autosomal flightless mutants of Drosophila melanogaster and identification of a new gene required for normal muscle structure and function. Genetics 137: 151–164. 8056306 10.1093/genetics/137.1.151 PMC1205932

[55] Vinckier A, Semenza G (1998) Measuring elasticity of biological materials by atomic force microscopy. FEBS Lett 430: 12–16. 9678586 10.1016/s0014-5793(98)00592-4

