# Supplementary figures and images for: DAAM Is Required for Thin Filament Formation and Sarcomerogenesis during Muscle Development in Drosophila
Source: PLoS Genet. 2014 Feb 27;10(2):e1004166. doi: 10.1371/journal.pgen.1004166 (PMC3937221; doi:10.1371/journal.pgen.1004166)

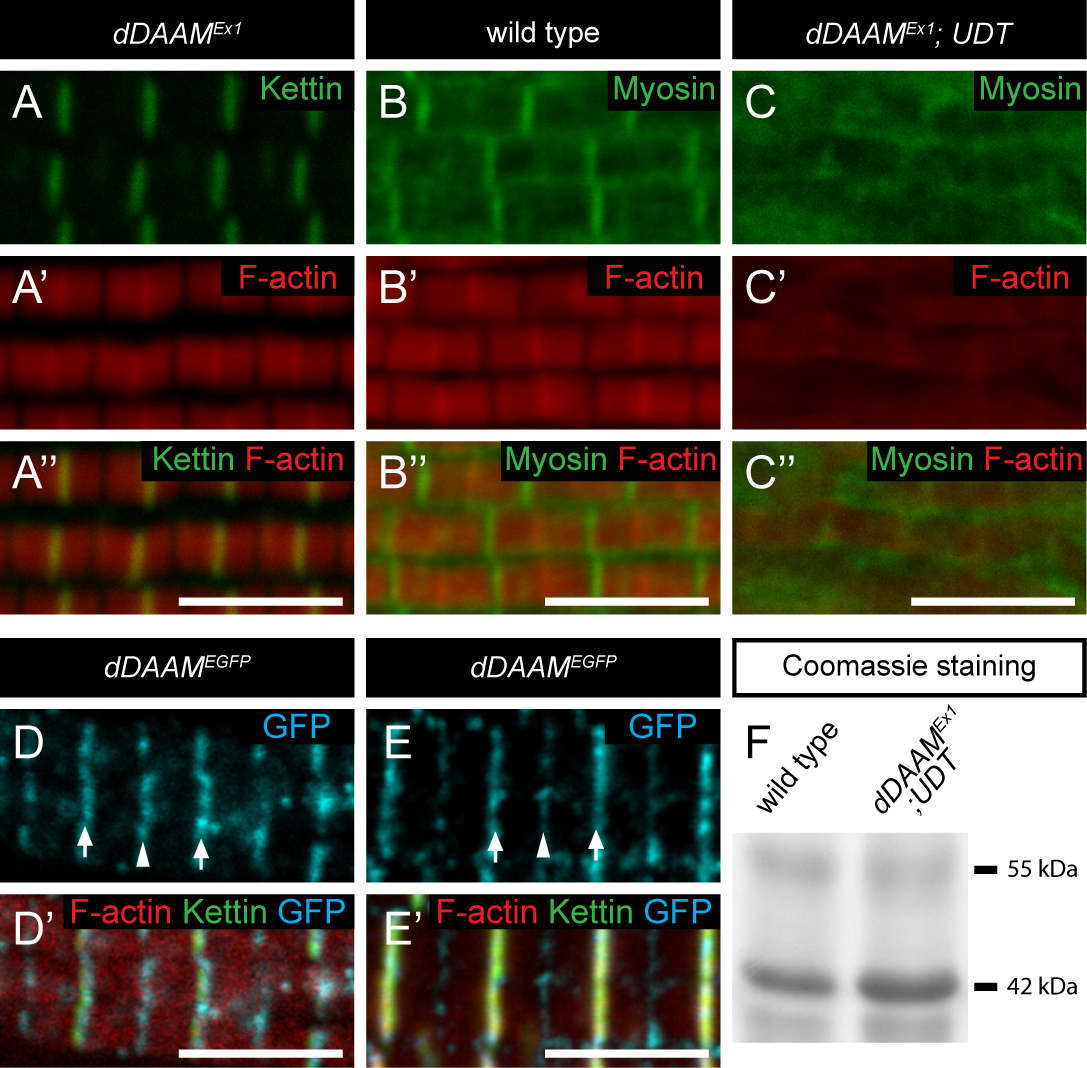

Supplement: Figure S1 — Impaired adult IFM structure in dDAAM mutants. (A–A″) IFM myofibrils of a flightless dDAAMEx1 mutant looks largely normal, although some of the sarcomeres show reduced lengths (2.5 µm instead of 3.2 µm; Kettin in green, actin in red). (B–C″) Myofibrils of wild type (B–B″) and dDAAMEx1, UDT mutants (C–C″) stained for Myosin (green) and actin (red). Note the severely impaired Myosin and M-line organization, and the strong reduction of F-actin level in IFM of the dDAAM mutant (C). In newly eclosed (D, D′) and 4 day-old (E, E′) dDAAMEGFP adults anti-GFP staining is evident at the Z-disc (arrows) and M-band (arrowhead). (F) Coomassie staining shows no significant difference in the amount of G-actin in wild type and dDAAMEx1, UDT mutants. Bars, 5 µm. (TIF) [file pgen.1004166.s001.tif]

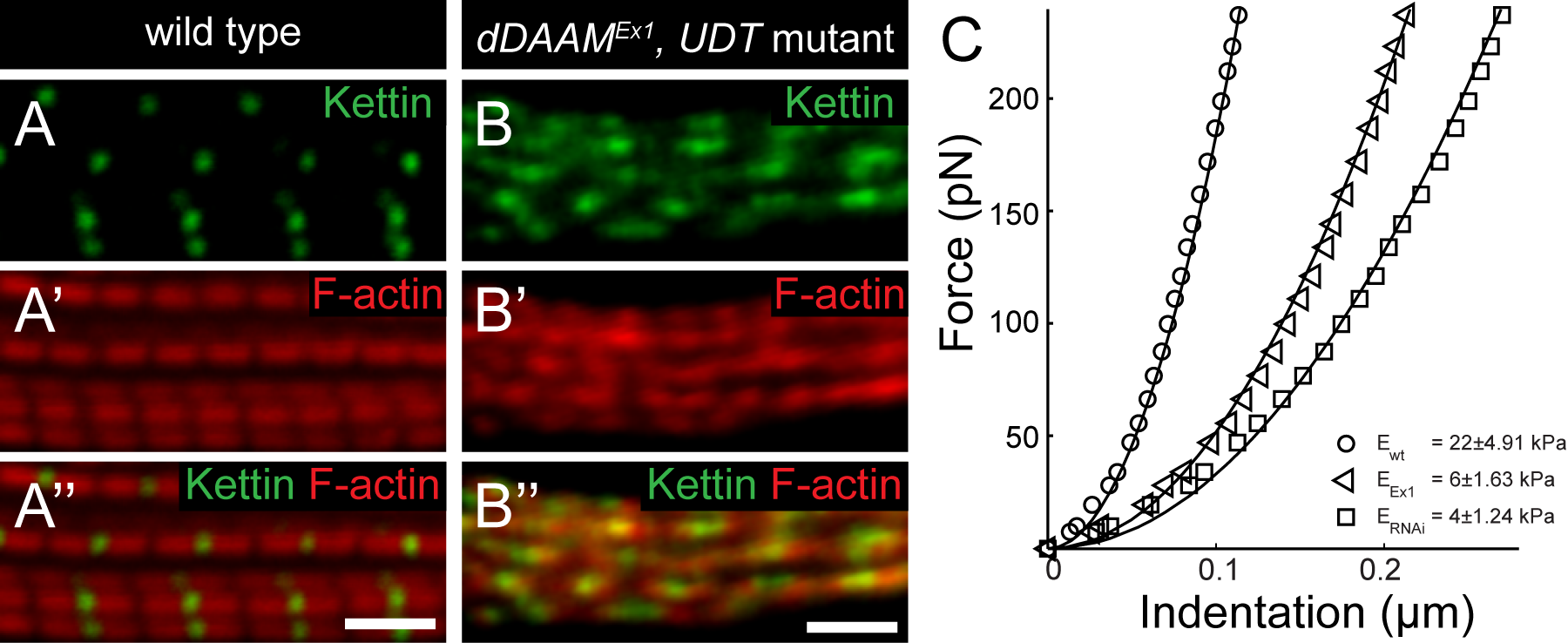

Supplement: Figure S2 — dDAAM impairs pupal IFM structure and the mechanical properties of muscles. Myofibrils from a wild type (A–A″) and dDAAMEx1, UDT mutant (B–B″) pupal IFM (48 hours APF, 29°C) stained for actin (in red) and Kettin (in green). The mutant IFM shows Z-disc and M-line organization defects. (C) Quantification of the transverse elasticity of wild type and dDAAM mutant myofibrils measured by Atomic Force Microscopy. To characterize the mechanical properties of the myofibrils, their transverse elasticity (Young's modulus) was calculated. The average curve is fitted with a second order polynomial (C). The elasticity of dDAAMEx1 and dDAAMEx1, UDT (RNAi) mutant fibers is significantly lower, 6±1.63 kPa (n = 35) and 4±1.24 kPa (n = 15), respectively, than the one of wild type, 22±4.91 kPa (n = 25). Bars, 2 µm. (TIF) [file pgen.1004166.s002.tif]

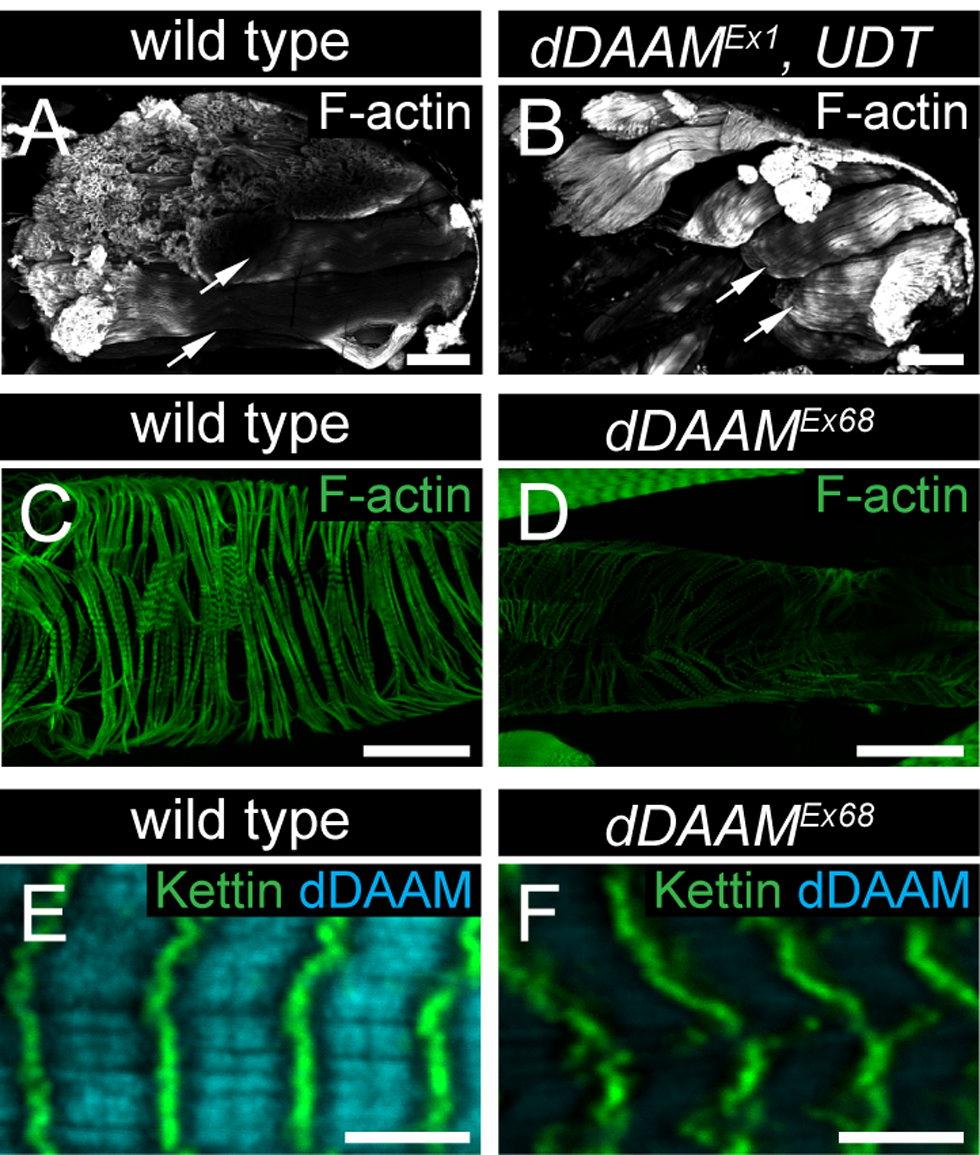

Supplement: Figure S3 — dDAAM affects IFM fiber morphology and heart tube development. (A–B) IFM structure of a wild type (A) and dDAAMEx1, UDT mutant (B) as seen under confocal microscope. In these sagittal sections of thoraces rhodamine-phalloidin was used to visualize the muscle F-actin. Note that mutant dorsolongitudinal muscle (DLM) fibers are shorter (arrows) and thinner than in wild type, and some of the muscles appear degenerated. (C–D) Phalloidin staining of a wild type (C) and dDAAMEx68 mutant (D) larval heart tube to visualize F-actin (in green). Compared to wild type, the dDAAMEx68 mutant has reduced F-actin levels, and heart tube diameter is smaller. In addition, many of the mutant myofibrils appear thinner than their wild type counterparts and often deviate from the typical wild type orientation. (E–F) A developing wild type (E) larval body wall muscle at 72 hours AEL clearly expresses and accumulates the dDAAM protein (in cyan) in its myofibrils. A similar but weaker dDAAM expression pattern can still be detected in ∼10% of dDAAMEx68 mutant larvae (F) even at 100 hours AEL. Kettin (in green) labels the Z-discs in E-F. Bars: 100 µm (A–B); 40 µm (C–D); 5 µm (E–F). (TIF) [file pgen.1004166.s003.tif]

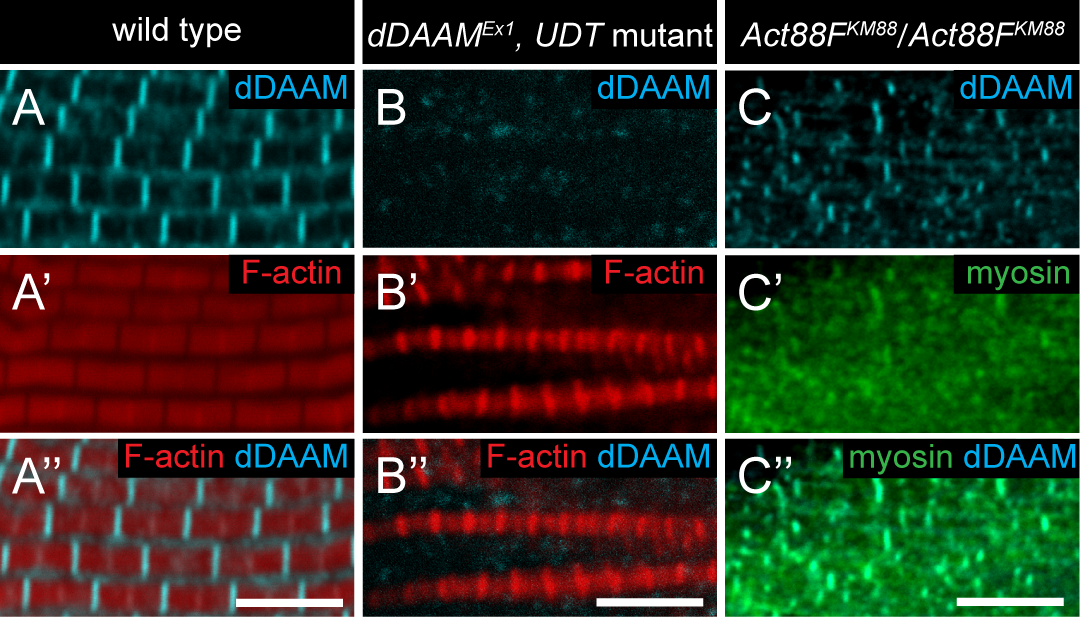

Supplement: Figure S4 — Sarcomeric localization of the dDAAM protein in wild type and mutant IFMs. Myofibrils of wild type (A–A″), dDAAMEx1, UDT (B–B″) and Act88F null mutant (C–C″) IFM from young adults stained for dDAAM (cyan, A–C″), actin (red, A′–B″) and Myosin (green, C′ and C″). Staining of wild type IFM reveals dDAAM accumulation at M-line and Z-disc, and in the sarcoplasm (A–A″). In contrast, in a dDAAMEx1, UDT mutant IFM only a weak background staining is evident (compare A to B). In Act88F null mutants, which completely lack sarcomeric thin filaments, dDAAM protein remains associated with muscle fibers and displays a partial colocalization with myosin (C–C″). Bars, 5 µm. (TIF) [file pgen.1004166.s004.tif]

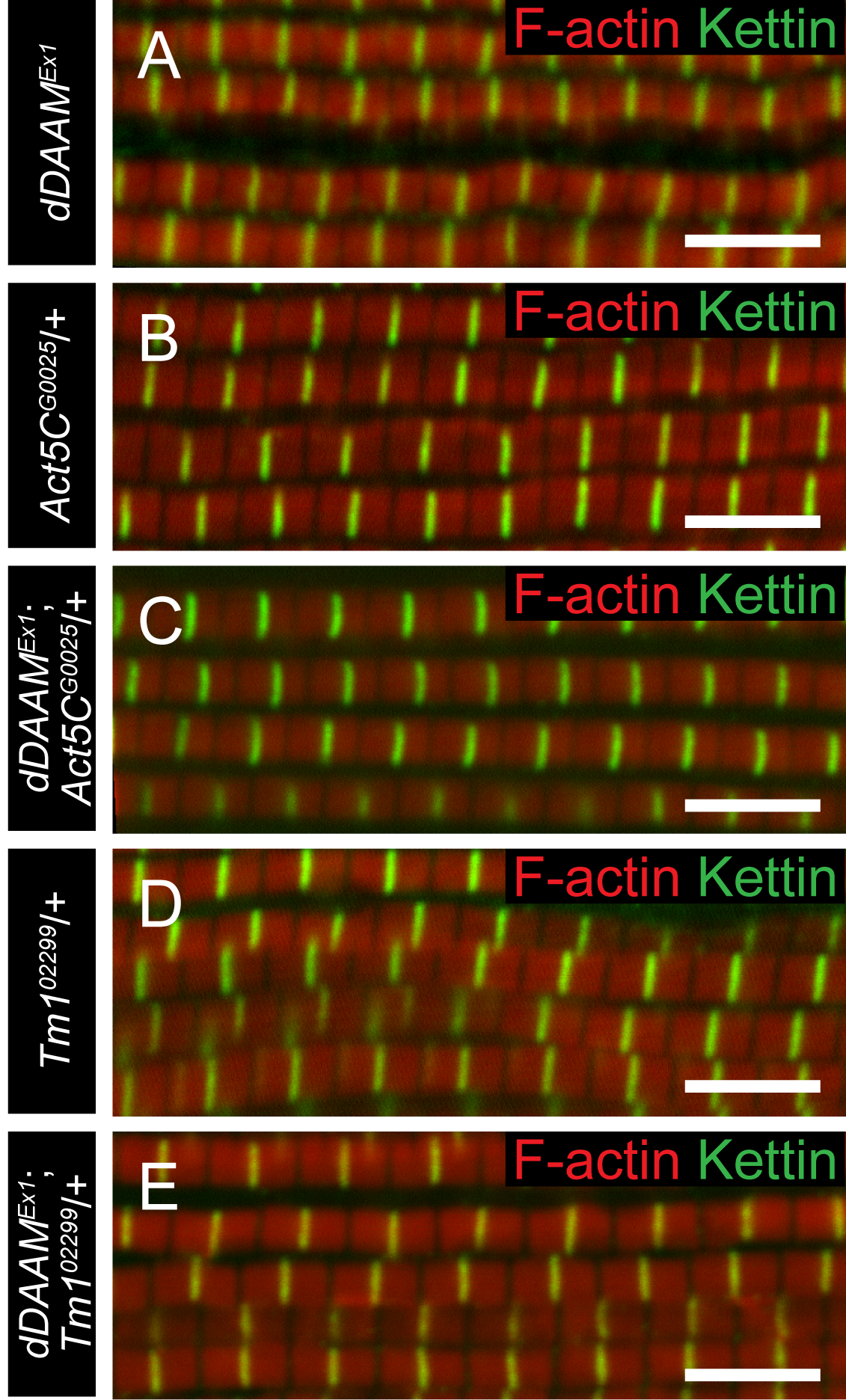

Supplement: Figure S5 — dDAAM shows no interaction with the non-muscle cell specific isoforms of actin and tropomyosin. Adult IFM myofibrils showing dDAAMEx1 (A), Act5CG0025/+ (B), dDAAMEx1; Act5CG0025/+ (C), Tm102299/+ (D) and dDAAMEx1; Tm102299/+ (E) mutants stained for Kettin (green) and actin (red). Note that all mutant myofibrils look nearly wild type. Bars, 5 µm. (TIF) [file pgen.1004166.s005.tif]
